# Inhibition of lipid synthesis by the HIV integrase strand transfer inhibitor elvitegravir in primary rat oligodendrocyte cultures

**DOI:** 10.3389/fnmol.2023.1323431

**Published:** 2023-12-11

**Authors:** Hubert Monnerie, Micah Romer, Lindsay M. Roth, Caela Long, John S. Millar, Kelly L. Jordan-Sciutto, Judith B. Grinspan

**Affiliations:** ^1^Department of Neurology, The Children’s Hospital of Philadelphia, Philadelphia, PA, United States; ^2^Institute of Diabetes, Obesity and Metabolism, University of Pennsylvania, Philadelphia, PA, United States; ^3^Department of Pathology, School of Dental Medicine, University of Pennsylvania, Philadelphia, PA, United States

**Keywords:** cART, Hand, HIV, myelin, oligodendrocyte, SREBP

## Abstract

Combined antiretroviral therapy (cART) has greatly decreased mortality and morbidity among persons with HIV; however, neurologic impairments remain prevalent, in particular HIV-associated neurocognitive disorders (HANDs). White matter damage persists in cART-treated persons with HIV and may contribute to neurocognitive dysfunction as the lipid-rich myelin membrane of oligodendrocytes is essential for efficient nerve conduction. Because of the importance of lipids to proper myelination, we examined the regulation of lipid synthesis in oligodendrocyte cultures exposed to the integrase strand transfer inhibitor elvitegravir (EVG), which is administered to persons with HIV as part of their initial regimen. We show that protein levels of genes involved in the fatty acid pathway were reduced, which correlated with greatly diminished *de novo* levels of fatty acid synthesis. In addition, major regulators of cellular lipid metabolism, the sterol regulatory element-binding proteins (SREBP) 1 and 2, were strikingly altered following exposure to EVG. Impaired oligodendrocyte differentiation manifested as a marked reduction in mature oligodendrocytes. Interestingly, most of these deleterious effects could be prevented by adding serum albumin, a clinically approved neuroprotectant. These new findings, together with our previous study, strengthen the possibility that antiretroviral therapy, at least partially through lipid dysregulation, may contribute to the persistence of white matter changes observed in persons with HIV and that some antiretrovirals may be preferable as life-long therapy.

## 1 Introduction

Worldwide, there are approximately 37 million people living with human immunodeficiency virus 1 (HIV-1). As a result of combined antiretroviral therapy (cART) introduced more than 25 years ago, HIV-associated mortality and morbidity have been dramatically reduced (Palella, 1998). Persons with HIV treated with cART are better able to achieve and maintain maximal and durable suppression of HIV-1 replication, resulting in survival rates that are close to a normal life span (Lohse and Obel, 2016). There are currently seven classes of approved antiretroviral drugs, based on how each drug interferes with the HIV life cycle. Guidelines from both the Centers for Disease Control (CDC) and the World Health Organization (WHO) recommend treating persons with HIV with an initial antiretroviral regimen that includes two nucleoside reverse transcriptase inhibitors (NRTIs), in combination with either a non-nucleoside reverse transcriptase inhibitor (NNRTI), a protease inhibitor (PI; boosted with cobicistat or ritonavir to increase bioavailability), or an integrase strand transfer inhibitor (INSTI) (World Health Organization, 2018; AIDSinfo, 2019).

Although cART is effective at reducing the viral load to undetectable levels, cognitive deficits are still a hallmark among persons with HIV, affecting up to 50% of patients (Eggers et al., 2017). HIV-associated neurocognitive disorders (HANDs) represent a spectrum of neurocognitive impairments, whose severity ranges from asymptomatic neurocognitive impairment (ANI), mild neurocognitive disorder (MND) to HIV-associated dementia (HAD). Although HAD prevalence has considerably declined in the post-cART era (<5%), milder forms of HAND remain elevated despite cART (Heaton et al., 2010). In fact, evidence that antiretroviral neurotoxicity may contribute to or increase the risk of neurologic deficits that persist in persons with HIV has been reported *in vitro*, *in vivo,* and in human subjects (Robertson et al., 2012; Akay et al., 2014; Shah et al., 2016; Stern et al., 2018). In particular, persons with HIV on INSTI-based regimens demonstrated worse learning/memory performance and smaller regional brain volumes compared with those on non-INSTI therapy (O’Halloran et al., 2019). Nevertheless, whether antiretroviral drug toxicity or HIV infection itself contributes to the persistence of HAND in persons with HIV, the mechanisms underlying these manifestations remain mostly undetermined.

The neuropathogenesis of persons with HIV includes white matter changes which remain prevalent in cART-treated persons with HIV (Heaton et al., 2011; Zhu et al., 2013). Furthermore, longer cART exposure is associated with lower white matter volumes in cART-treated persons with HIV (Jernigan et al., 2011). Structural abnormalities including size reduction of the corpus callosum are frequently observed despite cART and have been associated with neurocognitive impairments (Tate et al., 2011; Kelly et al., 2014; Buyukturkoglu et al., 2017). Indeed, there is a correlation between a higher risk of HAND and more abnormal white matter tracts (Muller-Oehring et al., 2010). Interestingly, transcriptomic analysis of brain tissues from HAND patients highlighted a subset of genes that remained dysregulated in response to antiretroviral treatment, including myelin-related genes that were downregulated in both untreated and treated persons with HIV, some being particularly suppressed in the white matter of persons with HIV on cART (Borjabad et al., 2011; Solomon et al., 2019).

In the CNS, myelin is synthesized by oligodendrocytes, and its role is essential for both CNS function and plasticity. While the neurotoxicity of antiretroviral drugs has drawn researchers’ attention, the effect of antiretroviral compounds on oligodendrocytes remains underexplored despite accumulating evidence of persistent myelin damage in persons with HIV on cART and its possible contribution to HAND. In light of this, we previously reported decreased oligodendrocyte maturation and reduced myelin protein levels both *in vitro* in mouse cultures and *in vivo* in mice following exposure to protease inhibitors at relevant therapeutic concentrations (Jensen et al., 2015). Furthermore, prefrontal cortex tissues from persons with HIV with HAND on cART had reduced myelin basic protein (MBP) levels compared with cART-naive persons with HIV or HIV-negative individuals (Jensen et al., 2015). More recently, we demonstrated that the INSTI elvitegravir (EVG), which belongs to a class of drugs currently used in first-line regimens prescribed to persons with HIV, prevented oligodendrocyte maturation *in vitro* and remyelination *in vivo* in rodents (Roth et al., 2020).

During myelination, oligodendrocytes synthesize a great amount of lipids that are incorporated into the myelin membrane, whose function is highly vulnerable to perturbations in lipid composition (Camargo et al., 2017; Monnerie et al., 2017). The expression of genes encoding enzymes essential for cholesterol and fatty acid synthesis is regulated by sterol regulatory element-binding proteins (SREBPs), transcription factors that are key regulators of cellular lipid homeostasis (Goldstein et al., 2006). There are three SREBP isoforms (SREBP-1a, SREBP-1c, and SREBP-2) that are synthesized as precursor proteins in the endoplasmic reticulum. In response to low sterol concentration, the sterol sensor SREBP-cleavage activating protein (SCAP) escorts the SREBP precursor protein to the Golgi, where it is sequentially cleaved by site-1 and site-2 proteases to release the mature form, which then translocates to the nucleus to activate genes involved in cholesterol and fatty acid synthesis and metabolism (Horton et al., 2002).

In the present study, we asked whether the integrase strand transfer inhibitor, elvitegravir (EVG), altered lipid metabolism in rat cerebral cortical oligodendrocyte cultures. We found that EVG dramatically altered SREBP protein levels, and this effect was accompanied by a diminution of fatty acid synthesis, which correlated with decreased protein levels of acetyl-CoA carboxylase (ACC) and fatty acid synthase (FASN), enzymes that are essential to fatty acid synthesis. Altered oligodendrocyte maturation manifested by a drastic reduction in the expression of a major myelin protein, proteolipid protein (PLP). In addition, EVG induced both a time- and dose-dependent increase in the phosphorylation of elF2α, indicating that the integrated stress response (ISR) was activated. Interestingly, most of EVG’s effects could be prevented by co-incubation with serum albumin, a clinically used neuroprotectant. These and our previous data strengthen the possibility that a subset of antiretroviral drugs from distinct antiretroviral classes may contribute to white matter damage in persons with HIV, which could help explain the persistence of HAND.

## 2 Materials and methods

### 2.1 Dissociated cerebral cortex oligodendrocyte cultures

All experiments were performed following the guidelines set forth by the Children’s Hospital of Philadelphia Institutional Animal Care and Use Committee (IACUC). Cerebral cortices were isolated from newborn Sprague–Dawley rats (Charles River Laboratories, Malvern, PA, United States, RRID: RGD_737891). Dams in their transport container were taken to the laboratory upon arrival, and the pups, at postnatal day 1 and of both sexes, were processed immediately for cell culture. The pups were euthanized by decapitation with frequently sharpened scissors. In general, the brains of two pups were pooled together before plating, for each cell culture. For all the experiments presented, approximately 170 pups were used. The dams were euthanized by CO_2_ inhalation following the euthanasia of the last pup in the litter. The cell isolation protocol has been previously described and was used with some modifications (Feigenson et al., 2009; Jensen et al., 2015). In brief, the tissue freed of meninges was minced and then incubated in 0.25% trypsin (Life Technologies, Grand Island, NY, United States, cat# 15090046, 2021) for 45 min at +37°C. Following tissue incubation, 10% fetal bovine serum (Sigma-Aldrich, St Louis, MO, United States, cat# F4135, 2020) was added, and the mixture was centrifuged at 500 g for 6 min. The supernatant was removed, Neurobasal medium (NBM; Life Technologies, cat# 21103049, 2021) supplemented with B27 (Life technologies, cat# 17504044, 2021) containing 0.5 mM L-glutamine (Life Technologies, cat# A2916801, 2021) and 5,000 U/mL Penicillin/Streptomycin (Life Technologies, cat# 15070063, 2020) was added, and the tissue was dissociated by trituration. The cell suspension was filtered through a 100 μm nylon mesh, and the cells were plated at 4 × 10^6^ cells/ml on poly-D-lysine (PDL; 10 μg/mL; Sigma-Aldrich, cat# P6407, 2021)-coated 100 mm cell culture dishes. After 24 h, the culture medium was replaced with growth medium (NBM/B27/L-glutamine/Penicillin/Streptomycin) containing 10 ng/mL basic fibroblast growth factor (bFGF; R&D Systems, Minneapolis, MN, United States, cat# 133-FB-025, 2020) and 2 ng/mL platelet-derived growth factor alpha (PDGFα; PeproTech, Rocky Hill, NJ, United States, cat# 100-13A, 2020). Cells were grown at +37°C in 5% CO_2_ and were fed growth medium every 2 days until they reached approximately 80% confluence.

Oligodendrocyte cultures were generated by using a modified washdown procedure (Feigenson et al., 2009). Cells were incubated with Ca^2+^/Mg^2+^-free Hank’s balanced salt solution (HBSS; Life Technologies, cat# 14175079, 2021) containing 0.025% trypsin/EDTA (Life Technologies, cat# 25200056, 2020) at +37°C for 3–4 min. Small, round process-bearing oligodendrocyte precursor cells (OPCs) were detached, leaving a population of cells still adhering to the dish. Then, cells were collected and centrifuged at 300 g for 5 min, and the pellet was resuspended in a growth medium. Cells were either plated at 1 × 10^5^ cells/ml on PDL-coated glass coverslips in 24-well culture plates or at 1 × 10^6^ cells/ml on PDL-coated 100 mm cell culture dishes. Cells were grown until they reached approximately 80% confluence and then were switched to the differentiation medium.

To differentiate OPCs into mature oligodendrocytes, the growth medium was replaced with the differentiation medium, consisting of DMEM/F12 (Life Technologies, cat# A4192001, 2021) supplemented with N2 (Life Technologies, cat# 17502001, 2021), containing 2 mM L-glutamine, 30% D-glucose, 0.4 μg/mL L-Thyroxine (T4; Sigma-Aldrich, cat# T0397, 2021), and 10 ng/mL biotin (Sigma-Aldrich, cat# B4639, 2020).

### 2.2 Determination of drug concentrations and drug treatments

The drug concentrations we used are based on previously reported plasma levels from human subjects, which were generally above 3 μM and below 10 μM, for both EVG and raltegravir (RAL) (Gilead Sciences International Limited, 2013; Podany et al., 2017; Ocque et al., 2018; Tsuchiya et al., 2018; Yonemura et al., 2018). Plasma concentrations of antiretrovirals may represent the higher end of drug concentration at the site of action and are much higher than those reported in patient cerebrospinal fluid (CSF) samples. However, brain tissue from humans, primates, and mice show higher antiretroviral concentrations than those reported in CSF (Srinivas et al., 2019; Ferrara et al., 2020). Additionally, low CSF concentrations may be compounded by decades-long brain exposure to antiretrovirals since cART requires a lifetime commitment. Our model’s objective was to assess the chronic impact of antiretrovirals, thus necessitating higher doses than that which may be observed in patients’ CSF at any measured time.

OPCs were placed into differentiation medium and exposed to elvitegravir (EVG; 3.5–10 μM; Toronto Research Chemicals, North York, ON, Canada, cat# E509000, 2020) or raltegravir (RAL; 3–10 μM; NIH AIDS Reagent Program, Germantown, MD, United States, cat# ARP-11680, 2019). EVG was prepared as a stock solution in DMSO and added directly to the culture medium. RAL was dissolved in PBS. Control cultures received an equivalent amount of DMSO or PBS. The integrated stress response (ISR) inhibitor ISRIB (5–20 μM; Sigma-Aldrich, cat# SML0843, 2020) was added to the cultures 2 h before EVG exposure. For treatment with recombinant human serum albumin, (HSA; 10 μM; Sigma-Aldrich, cat# A9731, 2021) HSA was solubilized in 50% PBS/50% DMEM/F12 and added to the cultures together with EVG. In some experiments, various dilutions (1:10,000–1:4,000) of an aqueous mixture of fatty acids (Sigma-Aldrich, cat # F7050, 2020) were added to the cells at the time of EVG treatment. OPCs were allowed to differentiate for 3 days before cells were processed.

### 2.3 Cell viability

Cell survival was assessed using fluorescein diacetate (Sigma-Aldrich, cat# F7378, 2019) and propidium iodide (Sigma-Aldrich, cat# P4170, 2019). Live and dead cells were determined by incubating with 15 μg/mL fluorescein diacetate and 4.5 μg/mL propidium iodide, respectively (15 min, +37°C). After several rinses in PBS, cells were counted in eight adjacent microscopic fields at 20X magnification, in each of the 3 wells per condition. Experiments were repeated three to four times. Cell survival was expressed as a percent of control values and represents a minimum of 1,000 cells counted per condition in each experiment.

### 2.4 Immunocytochemistry

Cells grown on coverslips were processed as live cells for cell surface antigen detection and were labeled with anti-A2B5, anti-O4, or anti-galactocerebroside (GalC), followed by several rinses in HBSS before secondary antibodies were added. After cells were rinsed multiple times, they were fixed in methanol for 4 min. The detection of internal antigens was performed as previously described (Feigenson et al., 2009; Jensen et al., 2015). Table 1 shows primary antibody details and use. In brief, for the detection of internal antigens, cells were fixed in methanol and washed in PBS, and non-specific sites were blocked with 10% normal goat serum (Sigma-Aldrich, cat# NS02L, 2019) in PBS for 30 min. Then, cells were incubated in 5% goat serum in PBS containing anti-proteolipid protein (PLP) rat hybridoma supernatant and rabbit anti-Olig2 (Sigma-Aldrich, cat# AB9610, RRID: AB_570666) for 1 h. After several PBS rinses, cells were incubated with fluorescein-conjugated goat anti-rat (1:260; Jackson Immunoresearch, West Grove, PA, cat# 112–095-062, RRID: AB_2338194) and Cy3-conjugated donkey anti-rabbit (1:900; Jackson Immunoresearch, cat# 711–165-152, RRID: AB_2307443) or rhodamine red-conjugated goat anti-rat (1,200; Jackson Immunoresearch, West Grove, PA, cat# 112–295-143, RRID: AB_2338293) or fluorescein-conjugated goat anti-mouse (1,200; Jackson Immunoresearch, West Grove, PA, cat# 115–545-020, RRID: AB_2338843) secondary antibodies diluted in 5% goat serum in PBS for 1 h, followed by several washes in PBS. Coverslips were mounted onto glass slides in DAPI-containing ProLong Gold Antifade mounting medium (Cell Signaling Technologies, Danvers, MA, United States, cat# 8961S, 2021). Oligodendrocytes were visualized, and fluorescent images were recorded with a Leica DM6000B fluorescence microscope and analyzed with the Leica LAS image analysis program.

**Table 1:** List of antibodies and primers.

Antibody	Host	Use and dilution	Source
A2B5	Mouse	ICC: 1:2	Hybridoma, Eisenbarth et al. (1979)
O4	Mouse	ICC: 1:4	Hybridoma, Sommer and Schachner (1981)
GalC	Mouse	ICC: 1:3	Hybridoma, Ranscht et al. (1982)
Olig2	Rabbit	ICC 1:900	Millipore-cat#AB9610, RRID:AB_570666
PLP	Rat	ICC: 1:2 WB 1:1,000	AA3, Yamamura et al., 1991
SREBP-1	Mouse	WB 1:150	Santa Cruz-cat#sc-13551, RRID:AB_628282
SREBP-2	Rabbit	WB 1:150	Abcam-cat#AB30682, RRID:AB_779079
FASN	Rabbit	WB 1:1,000	Cell Signaling-cat#3180, RRID:AB_2100796
ACC	Rabbit	WB 1:1,000	Cell Signaling-cat#3676, RRID:AB_2219397
HGMCR	Rabbit	WB 1:2,500	Thermo-Fisher-cat#PA5-37367, RRID:AB_2554032
α-tubulin	Mouse	WB 1:12,000	Sigma-Aldrich-cat#T5168, RRID:AB_477579
GAPDH	Mouse	WB 1:100,000	Millipore-cat#MAB374, RRID:AB_2107445
TBP	Mouse	WB 1:500	Millipore-cat#05-1531, RRID:AB_11212675
elF2α	Mouse	WB 1:1,000	Cell Signaling-cat#2103, RRID:AB_836874
Phospho-elF2α	Rabbit	WB 1:1,000	Cell Signaling-cat#9721, RRID:AB_330951
SCAP	Rabbit	WB 1:2,000	Bethyl-cat#A303-554A, RRID:AB_10953173

Target	Forward sequence	Reverse sequence
SREBP-1	5′-CCTGCTTGGCTCTTCTCTTT-3′	5′-CTGGTGCAGCTTATGGTAGAC-3′
SREBP-2	5′-GAGGCGGACAACACACAATA-3′	5′-CGG-CTCAGAGTCAATGGAATAG-3′
FASN	5′-GCTGCGGAAACTTCAGGAAAT-3′	5′-AGAGACGTGTCACTCCTGGACTT-3′
ACC	5′-CTTGTGGAATGCCTTGTGATTG-3	5′-CTGCTGCCGTCATAAGACAA-3′
PLP	5′-TAGGACATCCCGACAAGT-3′	5′-AAACAGGTGGAAGGTCATT-3′
PKG1	5′-ATGCAAAGACTGGCCAAGCTAC-3′	5′-AGCCA-CAGCCTCAGCATATTTC-3′

### 2.5 Western blots

For whole cell extracts, cells were harvested in cold 25 mM Tris (pH 7.4), 1 mM EDTA, 1% SDS, 1% Triton X-100, and 150 mM NaCl containing protease and phosphatase inhibitor cocktails (Roche Diagnostics, Indianapolis, IN, United States, cat# 11836170001, cat# 04906837001, 2021), sonicated, and then centrifuged 20 min at 14,000 rpm. Cytosolic and nuclear extracts for detection of SREBP proteins were prepared by harvesting the cells in cold 10 mM HEPES, 10 mM NaCl, 10 mM EDTA, 1 mM dithiothreitol, and 4% Triton X-100 containing protease and phosphatase inhibitors. Lysates were centrifuged for 5 min at 14,000 rpm, and the supernatant (cytosolic fraction) was collected and then stored at -80°C. To extract nuclear proteins, the pellet was resuspended in cold high salt buffer containing 20 mM HEPES, 400 mM NaCl, 1 mM EDTA, 10% glycerol, and 1 mM dithiothreitol with protease and phosphatase inhibitors. Nuclear proteins were incubated on a rocking platform (200 rpm) for 2 h at +4°C. The debris was pelleted by centrifugation at 14,000 rpm, and the supernatant, corresponding to the nuclear fraction, was collected and stored at -80°C. Protein amounts were determined by BCA protein assay (Pierce, Rockford, IL, United States, cat# 23227, 2021). Equal protein amounts were diluted in NuPAGE LDS buffer (Life Technologies, cat# NP0007, 2021) containing 2.5% β-mercaptoethanol and separated on NuPAGE Bis-Tris (Life Technologies, cat# NP0322BOX, 2021) or Tris-Acetate (Life Technologies, cat# EA0375BOX, 2021) gels and subsequently transferred to nitrocellulose membranes. After blocking in TBST (10 mM Tris, pH 8.0, 150 mM NaCl, 0.05% Tween 20) containing 5% non-fat dry milk, the membranes were incubated with primary antibodies in TBST/5% bovine serum albumin (BSA) overnight at 4°C (Table 1). When anti-PLP was used, NuPAGE gels were run under non-reducing conditions. Washed nitrocellulose membranes were incubated with horseradish peroxidase (HRP)-linked donkey anti-rabbit IgG (1:80,000–140,000; GE Healthcare, Piscataway, NJ, United States, cat# NA934, RRID:AB_772206) or HRP-linked sheep anti-mouse IgG (1:30,000–50,000; GE Healthcare, cat# NA931, RRID:AB_772210) in TBST/5% BSA for 1 h at RT. Proteins were visualized using SuperSignal West Dura Extended Duration Substrate (Thermo Scientific, Rockford, IL, United States, cat# 34075, 2021) and exposed to HyBlot CL films (Thomas Scientific, Swedesboro, NJ, United States, cat# 1141 J51, 2020). In some experiments, washed nitrocellulose membranes were incubated with IRDye 800CW goat anti-mouse IgG (1:12,000; Li-Cor, Lincoln, NE, United States, cat# 926–32,212, RRID:AB_621847), IRDye 680RD goat anti-rabbit IgG (1:12,000, Li-cor, cat# 926–68,071, RRID:AB_10956166), or IRDye 800CW goat anti-rat IgG (1:12,000, Li-Cor, cat# 926–32,219, RRID:AB_1850025) in TBST/5% BSA for 1 h at RT. Then, protein bands were visualized and quantified using the Odyssey infrared imaging system (Li-Cor). To quantify protein bands on film, a scanned image was analyzed on a Macintosh computer using the public domain Java image processing and analysis program NIH ImageJ 1.51 V (RRID:SCR_003070) inspired by the NIH Image developed at the US National Institute of Health and available on the Internet at https://imagej.nih.gov/ij/. Band intensities were normalized using anti-α-tubulin (Sigma-Aldrich, cat# T5168, RRID:AB_477579), anti-glyceraldehyde 3-phosphate dehydrogenase (GAPDH, Sigma-Aldrich, cat# MAB374, RRID:AB_2107445), or TATA box-binding protein (TBP, Sigma-Aldrich, cat# 05–1,531, RRID:AB_11212675) signals and then calculated as a percentage of control bands in the same membrane. Experiments were repeated at least three times for data analysis.

### 2.6 Quantitative reverse transcription polymerase chain reaction (qRT-PCR)

Cells were harvested after 3 days of differentiation. Total RNA was extracted with TRIzol (Molecular Research Center, Cincinnati, OH, United States, cat# 15596026, 2020) and then purified using the RNeasy Mini Kit (Qiagen, Valencia, CA, United States, cat# 74104, 2020). Three micrograms of purified RNA were converted to cDNA using the Superscript III First-strand Kit (Life Technologies, cat# 18080–051, 2020), and 80 ng of cDNA were used to perform qRT-PCR using Power SYBR Green as previously described (French-Morein et al., 2009; Feigenson et al., 2011). Primer sequences are listed in Table 1. Samples were measured in triplicate for each experiment from three biological replicates. Data were normalized using phosphoglycerate kinase 1 (PGK1) and were analyzed according to the comparative threshold (ΔΔCT) cycle method. All experiments were repeated at least three times using independent biological replicates that were treated independently for data analysis.

### 2.7 ^13^C-sodium (^13^C-NaAc) acetate labeling and quantification of *de novo* cholesterol and fatty acid synthesis

Cells in a differentiation medium were incubated with 1 mM ^13^C-NaAc-containing labeling medium (Cambridge Isotope Laboratories, Andover, MA, United States, cat# CLM-156-1) for the last 24 h of the 3-day differentiation paradigm before they were harvested. Following harvest, D_7_-cholesterol and heptadecanoate were added to cells as internal standards for cholesterol and fatty acids, respectively. Samples were saponified with KOH (0.3 M in ethanol), dried, and then incubated with BF_3_ (15% in methanol) at +70°C for 30 min to derivatize fatty acids. The reaction was stopped with water, and the lipid fraction was extracted into hexane, dried under nitrogen, and then incubated at room temperature for 30 min with pentafluorobenzoyl chloride (2.2% final concentration) in toluene-containing pyridine (8.8% final concentration) as a catalyst to derivatize cholesterol. The reaction was stopped by the addition of water, and the fraction containing derivatized cholesterol and fatty acids was extracted into petroleum ether. ^13^C-acetate enrichment in media was determined as described by Tomcik et al. (2011). In brief, methanol was added to the media to precipitate any protein, and the deproteinized sample was then incubated with 100 mM PFBBr solution in acetone. Samples were incubated at +65°C for 1 h, and then, the derivatized acetate was extracted into hexane.

All isotope enrichment measurements were determined by the IDOM Metabolic Tracer Resource at the University of Pennsylvania using gas chromatography/mass spectrometry using an Agilent 7890A/5975 system. The acetate ^13^C-enrichment was determined using negative chemical ionization (monitoring m/z 59–60). The cholesterol ^13^C-enrichment was determined using negative chemical ionization (monitoring m/z 580–588). The cholesterol peak area was normalized to the internal standard, and the concentration was determined using a standard curve. The percent of newly made cholesterol was determined using the equation:


$$\begin{array}{l} \%\mathrm{newly made cholesterol}=\\ \;\;\;\;\;\;\;\;\;\;\;\;\;\;\;[{\mathrm{total}}^{13}C-\mathrm{cholesterol enrichment}\;\left(\%\right)/\\ \;\;\;\;\;\;\;\;\;\;\;\;\;\;{(}^{13}C-\mathrm{acetate enrichment}\;\left(\%\right)\times n_{c})]\times 100 \end{array}$$

where *n_c_* is the number of labeled acetate precursors incorporated into cholesterol, assumed to equal 12 (Kelleher et al., 1994). The absolute amount of cholesterol synthesized is determined by multiplying the % newly made cholesterol by the corresponding cholesterol concentration. The palmitate ^13^C-enrichment was determined by using electron impact ionization (monitoring m/z 270–271 for palmitate and m/z 284 for the heptadecanoate internal standard). The palmitate peak area was normalized to the internal standard, and the concentration was determined using a standard curve. The rate of lipid synthesis was determined as the percent contribution of newly made palmitate, using the equation:


$$\begin{array}{l} \%\mathrm{newly made palmitate}=\\ \;\;\;\;\;\;\;\;\;\;\;\;\;\;\;\;\;\;\;\;\;\;\;\;[{\mathrm{total}}^{13}C-\mathrm{palmitate enrichment/}\\ \;\;\;\;\;\;\;\;\;\;\;\;\;\;\;\;\;\;\;\;\;\;\;\;{(}^{13}C-\mathrm{acetate enrichment}\;\left(\%\right)\times n_{p}\mathrm{)]}\times 100 \end{array}$$

where *n_p_* is the number of labeled acetate precursors incorporated into palmitate, assumed to equal 8 (Lee et al., 1995). The absolute amount of newly made palmitate was determined by multiplying the % newly made palmitate by the concentration of palmitate. The final cholesterol and palmitate values were normalized against the total protein amount present in each sample for comparison among different groups.

### 2.8 Palmitic acid (PA) preparation

We used BSA as a carrier to solubilize palmitate (PA) in aqueous solution. First, we prepared a 2 mM solution of sodium palmitate (Sigma-Aldrich, cat# P9767, 2020) in PBS by heating at +70°C with constant stirring until a clear liquid was obtained. Then, a 0.4 mM solution of BSA in DMEM/F12 was made and filtered at 0.22 μm. The conjugation of PA and BSA was achieved by mixing an equal volume of each solution under continuous agitation for 1 h at +37°C. This corresponded to a 5:1 molar ratio of PA:BSA. The BSA-conjugated PA is stored at −20°C until used. On the day of the experiment, the PA/BSA solution is incubated at +37°C for at least 30 min with gentle shaking before use. Cells were either treated with the PA/BSA solution at a final concentration of 50 μM or a control solution containing only PBS/BSA at the same dilution.

### 2.9 Statistical analysis

No inclusion or exclusion criteria were pre-determined in this study. No assessment of the normality of data or test for outliers was conducted. No data points were excluded. Statistical analyses and sample size were based on our previously published report (Monnerie et al., 2017), and the effect size was calculated for each individual data set.

Statistical analysis was performed with Microsoft Excel^TM^ 2016 Analysis ToolPak. The values are expressed as mean ± SEM. Measurements from at least three independent cell culture preparations were combined. For statistical comparisons of protein band immunoreactivities in Western blot experiments and analysis of qRT-PCR data, an unpaired two-tailed Student’s *t*-test was performed. In some experiments, GraphPad Prism was used to compare control and treated groups by one-way analysis of variance (ANOVA) followed by Fisher’s least significant difference post-hoc test for pairwise comparisons. The significance level was *p* < 0.05.

## 3 Results

### 3.1 EVG induces cell loss in a dose-dependent manner

**Figure 1:**
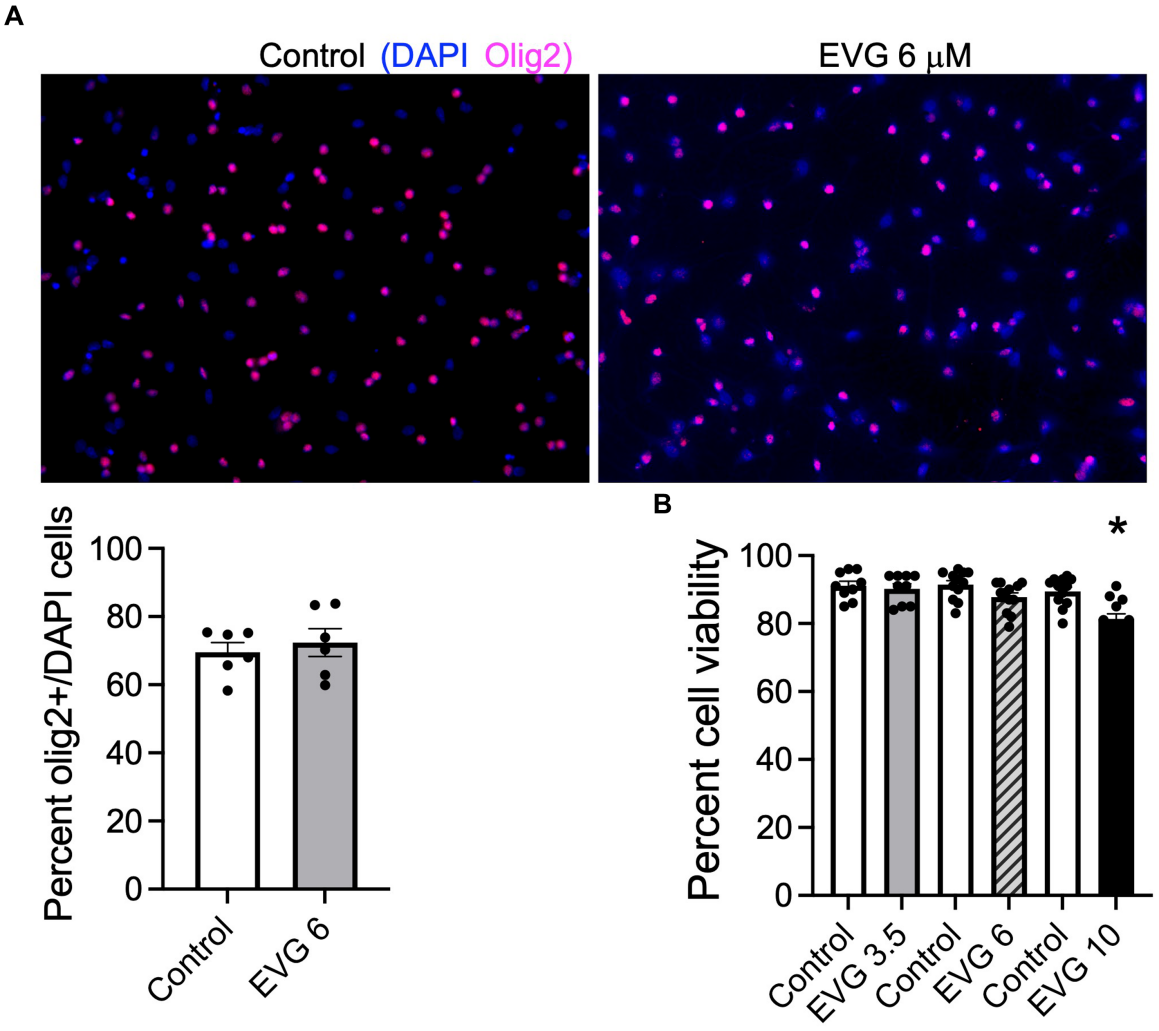
Effect of elvitegravir (EVG) on cell viability in oligodendrocyte cultures. Oligodendrocyte cultures were grown in a differentiation medium with EVG (3.5, 6, or 10 μM) or without (Control). After 3 days, cells were subjected to immunocytochemistry. **(A)** Representative images of Olig2 immunostaining in oligodendrocyte cultures. Cells were co-labeled for DAPI to stain nuclei (blue) and Olig2 to label oligodendrocyte lineage cells (pink). Scale bar: 50 μm. The graph represents the number of Olig2-positive cells normalized to DAPI. Data are expressed as mean ± SEM from 2 independent cell culture preparations. **(B)** Cell viability measured as the ratio of live (fluorescein diacetate-positive)/dead (propidium iodide-positive) + live cells. The graph represents the percent cell viability after exposure to various EVG concentrations. Data are expressed as mean ± SEM from four independent cell culture preparations. *p < 0.001 versus control.

To determine whether EVG exposure affected cell survival in our system, OPC cultures (enriched to _˜_70%, Figure 1A) grown with growth factors were induced to differentiate into mature oligodendrocytes after growth factors were removed and cells were placed in a differentiation medium. Cells were incubated with low (3.5 μM), intermediate (6 μM), and high (10 μM) EVG concentrations when switched to a differentiation medium and allowed to differentiate for 3 days before cell viability was assessed by quantifying the number of live/dead cells in both EVG-treated and control cultures. At 3.5 μM EVG, cell viability was not affected. Cells exposed to 6 μM EVG showed a 4% decrease in cell viability compared with controls, whereas 10 μM EVG significantly decreased cell viability by approximately 10% (Figure 1B). Since a significant cell loss was observed with 10 μM EVG, subsequent experiments analyzing its effect on oligodendrocyte cultures were conducted with the low (3.5 μM) and intermediate (6 μM) doses only.

### 3.2 EVG impairs differentiation of oligodendrocytes

**Figure 2:**
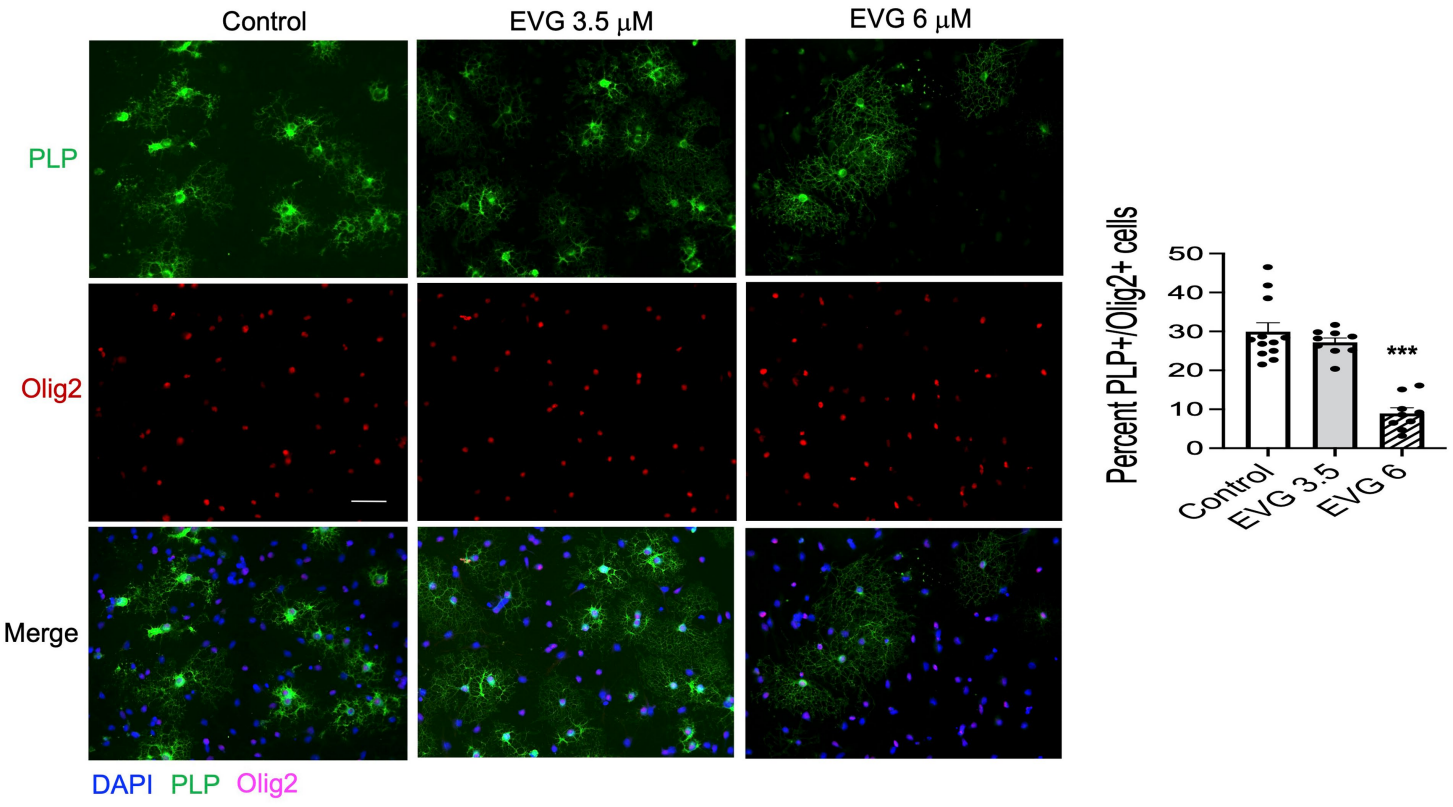
Elvitegravir (EVG) decreases oligodendrocyte maturation *in vitro*. Oligodendrocyte cultures were grown in a differentiation medium with EVG (3.5 or 6 μM) or without (Control). After 3 days, cells were subjected to immunocytochemistry. Representative images of proteolipid protein (PLP) immunostaining in oligodendrocyte cultures. Cells were triple-labeled for DAPI to stain nuclei (blue), PLP to label mature oligodendrocytes (green), and Olig2 to label oligodendrocyte lineage cells (pink). Scale bar: 50 μm. The graph represents the number of PLP-positive cells normalized to the number of Olig2-expressing cells. Data are expressed as mean ± SEM from three to four independent cell culture preparations. ***p < 0.0001 versus control (ANOVA).

**Figure 3:**
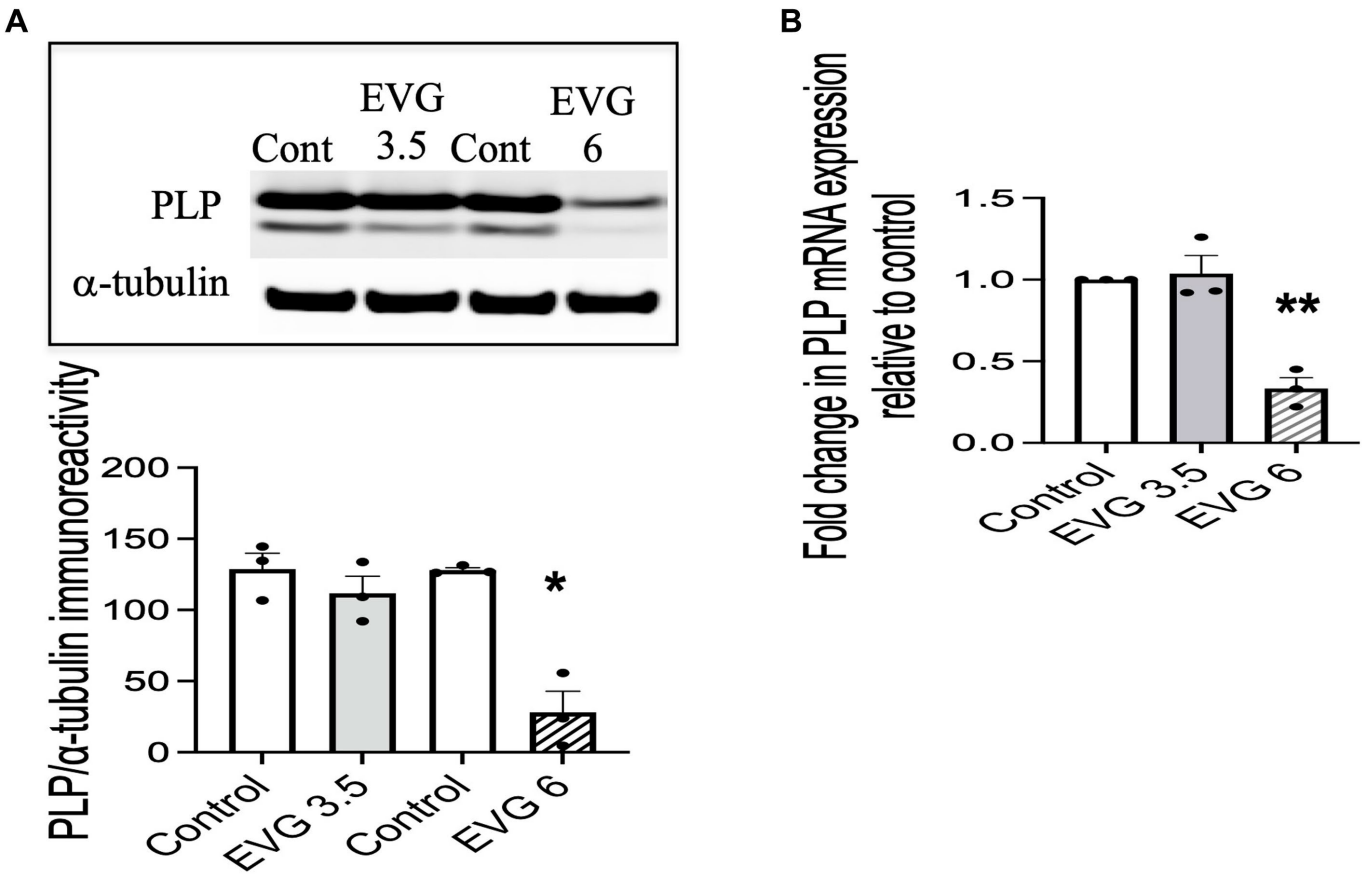
Elvitegravir (EVG) decreases myelin protein formation in oligodendrocytes *in vitro*. Oligodendrocyte cultures were grown in a differentiation medium with EVG (3.5 or 6 μM) or without (Control). After 3 days, cells were harvested and prepared for immunoblotting or qRT-PCR. **(A)** Cell lysates were immunoblotted for PLP, and band intensities were quantified after normalization to α-tubulin as loading control. Graph data are expressed as mean ± SEM from three independent cell culture preparations. *p < 0.05 versus control. **(B)** qRT-PCR analysis was performed to determine PLP mRNA expression relative to that of control. Graph data are expressed as mean ± SEM from three independent cell culture preparations. **p < 0.01 versus control.

Proteolipid protein (PLP) is a major myelin protein that is highly expressed in mature oligodendrocytes. To determine the effect of EVG treatment on the mature oligodendrocytes, OPC cultures were differentiated for 3 days with or without EVG (3.5 or 6 μM) before assessing the number of PLP- and Olig2-positive cells in EVG-treated and control cultures. As shown in Figure 2, the number of PLP-expressing cells was significantly lower in cultures treated with 6 μM EVG compared with controls, whereas 3.5 μM EVG did not significantly alter PLP-positive cell numbers. The ratio of Olig2-positive/DAPI-positive cell numbers remained unaltered regardless of treatment, distinguishing the effect of EVG on oligodendrocyte differentiation versus survival. In addition, Western blot analysis of cell extracts after 3 days of differentiation confirmed that treatment with 6 μM EVG caused drastically lower PLP levels compared with controls, whereas 3.5 μM did not (Figure 3A). This effect paralleled the lower PLP mRNA levels observed with 6 μM EVG (Figure 3B).

To determine whether EVG-exposed oligodendrocytes arrested at a specific stage during differentiation or remained as OPCs, cells differentiated for 3 days were immunolabeled with A2B5, a marker of OPCs (Eisenbarth et al., 1979), anti-O4, a late progenitor antigen (Sommer and Schachner, 1981), and anti-galactocerebroside (GalC), an immature oligodendrocyte marker (Ranscht et al., 1982). While the number of A2B5-labeled OPCs remained unchanged after 3 days of exposure to 6 μM EVG and that of O4-expressing cells was decreased by 30% following EVG treatment compared with controls, the reduction in GalC-positive cell number was 40% (Table 2). Furthermore, the number of PLP-expressing cells was 70% less in cultures treated with 6 μM EVG compared with controls. Together, these data suggest that while EVG does not block the onset of differentiation, its inhibitory effect is more pronounced at later stages of maturation.

**Table 2:** Effect of EVG (6 μM) at various stages of oligodendrocyte differentiation.

Antigen	% expressing cells	% change versus control	*df*	*t*-value	*P*-value
A2B5	Control: 52.8 ± 3.4				
	EVG: 47.9 ± 3.3	No change	16	1.04	*P* > 0.05
O4	Control: 38.1 ± 1.5				
	EVG: 26.7 ± 1.9	−30%	16	4.62	*P* < 0.0005
GalC	Control: 32.6 ± 2.6				
	EVG: 18.2 ± 3.1	−40%	16	3.57	*P* < 0.005
PLP	Control: 29.9 ± 8.6				
	EVG: 8.9 ± 3	−70%	18	7.74	*P* < 0.0001

### 3.3 EVG inhibits fatty acid synthesis

**Figure 4:**
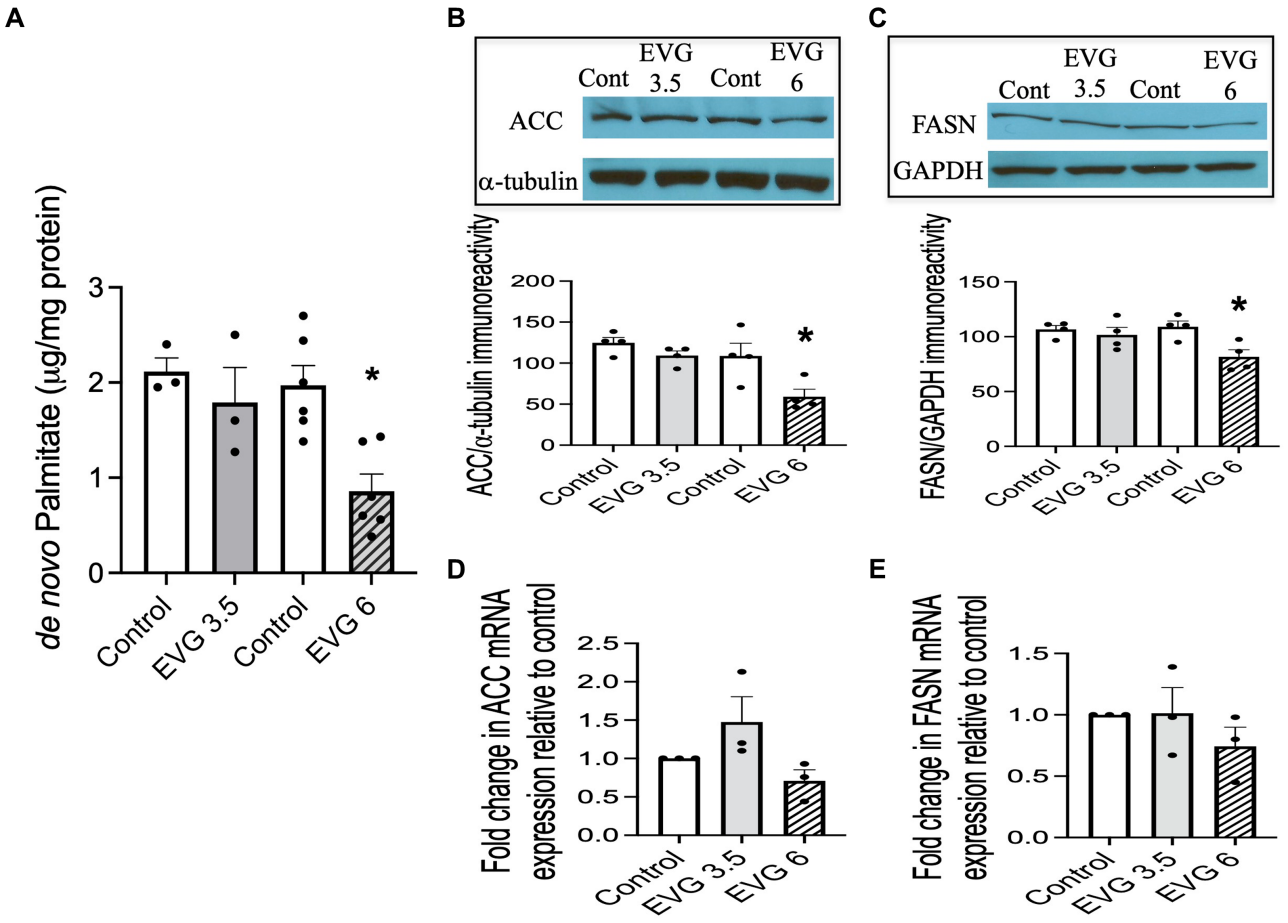
Effect of elvitegravir (EVG) on fatty acid synthesis and protein expression levels of key enzymes in the fatty acid pathway. Oligodendrocyte cultures were grown in a differentiation medium with EVG (3.5 or 6 μM) or without (Control) for 3 days. **(A)** Twenty-four hours before harvesting, cells were incubated with 1 mM ^13^C-sodium acetate. Cells were harvested and processed for quantification of *de novo* palmitate synthesis by gas chromatography/mass spectrometry. The graph represents new palmitate synthesis normalized to the total amount of proteins. Data are expressed as mean ± SEM from three to six independent cell culture preparations. *p < 0.005 versus control. **(B,C)** Western blot analysis and quantification of band intensities of **(B)** acetyl CoA carboxylase (ACC) and **(C)** fatty acid synthase (FASN) protein immunoreactivities normalized to GAPDH or α-tubulin as loading controls. Graph data are expressed as mean ± SEM from three to four independent cell culture preparations. *p < 0.05 versus control. **(D,E)** qRT-PCR analysis to determine **(D)** ACC and **(E)** FASN mRNA expression relative to that of control. Graph data are expressed as mean ± SEM from three independent cell culture preparations.

A major biochemical characteristic of the highly specialized myelin membrane is its high lipid-to-protein ratio, with lipids accounting for at least 70% of the dry weight of the membrane (Norton and Poduslo, 1973). This high lipid content is critical to myelin’s function. Since exposure to EVG impaired oligodendrocyte differentiation, we asked whether fatty acid synthesis was also adversely affected, given that most myelin lipids are structurally fatty acid-based. We quantified *de novo* synthesis of palmitate (PA) by employing ^13^C-sodium acetate labeling of lipids and mass spectrometry. While PA synthesis was not affected by 3.5 μM EVG, it was dramatically reduced by approximately 60% with 6 μM EVG, compared with controls (Figure 4A).

### 3.4 The protein expression of key enzymes in the fatty acid pathway is reduced following EVG exposure

Next, we examined the expression of acetyl-CoA carboxylase (ACC) and fatty acid synthase (FASN) in oligodendrocyte cultures differentiated for 3 days with or without EVG (3.5 or 6 μM) since both enzymes are essential for fatty acid synthesis. We found that both protein levels were significantly reduced by approximately 40 and 20%, respectively, at 6 μM EVG compared with controls (Figures 4B,C). In contrast, mRNA expression levels for ACC and FASN were not significantly different between controls and EVG-treated cells (Figures 4D,E). Thus, EVG exposure to oligodendrocyte cultures results in perturbation of fatty acid metabolism.

### 3.5 The sterol regulatory element-binding protein-1 (SREBP) protein and mRNA expressions are altered in the presence of EVG

The SREBPs are major regulators of cellular lipid metabolism, in particular, SREBP-1, which preferentially increases the transcription of genes involved in fatty acid and triglyceride synthesis. Therefore, we determined whether EVG affected SREBP-1 processing by assessing the respective levels of membrane-bound (precursor) and nuclear (mature) forms of SREBP-1 in oligodendrocyte cultures by Western blotting.

**Figure 5:**
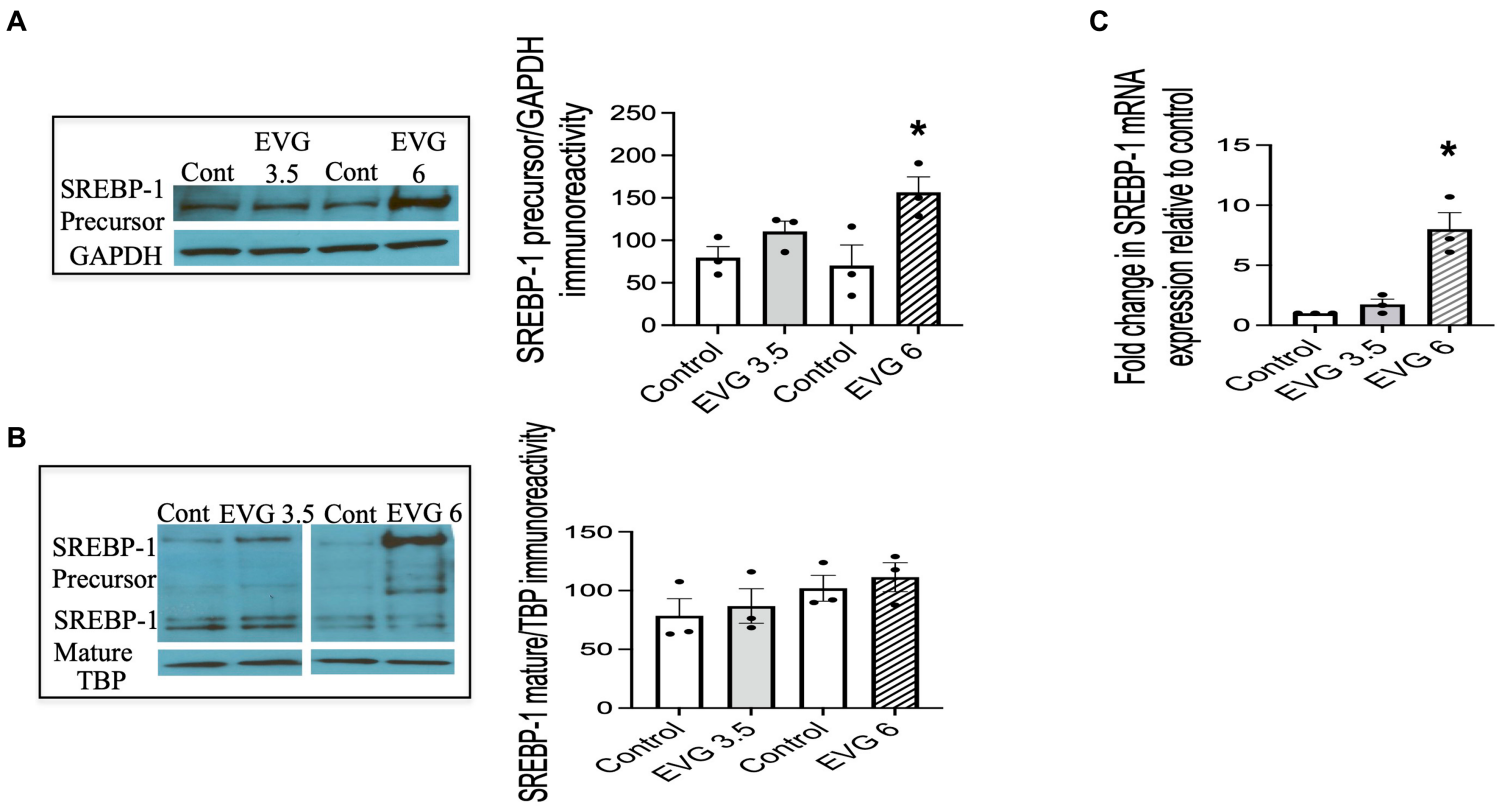
EVG alters the protein and mRNA expressions of sterol regulatory element-binding protein 1 (SREBP-1) in oligodendrocyte cultures. Oligodendrocyte cultures were grown in a differentiation medium with EVG (3.5 or 6 μM) or without (Control). After 3 days, cells were harvested and prepared for immunoblotting or qRT-PCR. **(A,B)** Western blots and quantification of band intensities of SREBP-1 precursor **(A)** and mature form **(B)** normalized to glyceraldehyde-3-phosphate dehydrogenase (GAPDH) or TATA box-binding protein (TBP) as loading controls. Graph data are expressed as mean ± SEM from three independent cell culture preparations. *p < 0.05 versus control. In **(C)**, the graph shows mRNA expression for SREBP-1 relative to the control. Graph data are expressed as mean ± SEM from three independent cell culture preparations. *p < 0.05 versus control.

We previously established that OPCs and differentiating oligodendrocytes expressed both SREBP-1 and SREBP-2 in culture (Monnerie et al., 2017). OPC cultures were switched to a differentiation medium and incubated with or without EVG (3.5 or 6 μM) for 3 days and harvested for analysis. Figure 5 shows that SREBP-1 protein levels were markedly increased at 6 μM compared with controls (Figure 5A), whereas no significant difference was observed at 3.5 μM EVG. In contrast, EVG exposure did not change the expression of the SREBP-1 mature form at any of the concentrations tested (Figure 5B). Increased levels of SREBP-1 precursor form were detected in whole cell extracts (Figure 5A) and cytoplasmic fractions (data not shown), as expected for an endoplasmic reticulum membrane-bound protein, but were also observed in the nuclear fraction following EVG exposure (Figure 5B). Fraction purity assessed by Western blotting using antibodies against nuclear TATA box-binding protein (TBP) and cytosolic glyceraldehyde 3-phosphate dehydrogenase (GAPDH) did not detect cross-contamination between the nuclear and cytosolic fractions. This observation is not unusual as overexpression of the SREBP-1 gene in HEK 293 cells similarly resulted in substantial amounts of precursor form in the nuclear fraction (Hua et al., 1996).

Since SREBPs can activate the transcription of their own genes, we examined whether EVG also affected transcript levels of SREBP-1. The results from qRT-PCR analysis performed on differentiating oligodendrocyte cultures for 3 days showed that SREBP-1 mRNA expression was also dramatically increased in the presence of 6 μM EVG (Figure 5C).

### 3.6 EVG does not impair cholesterol synthesis

**Figure 6:**
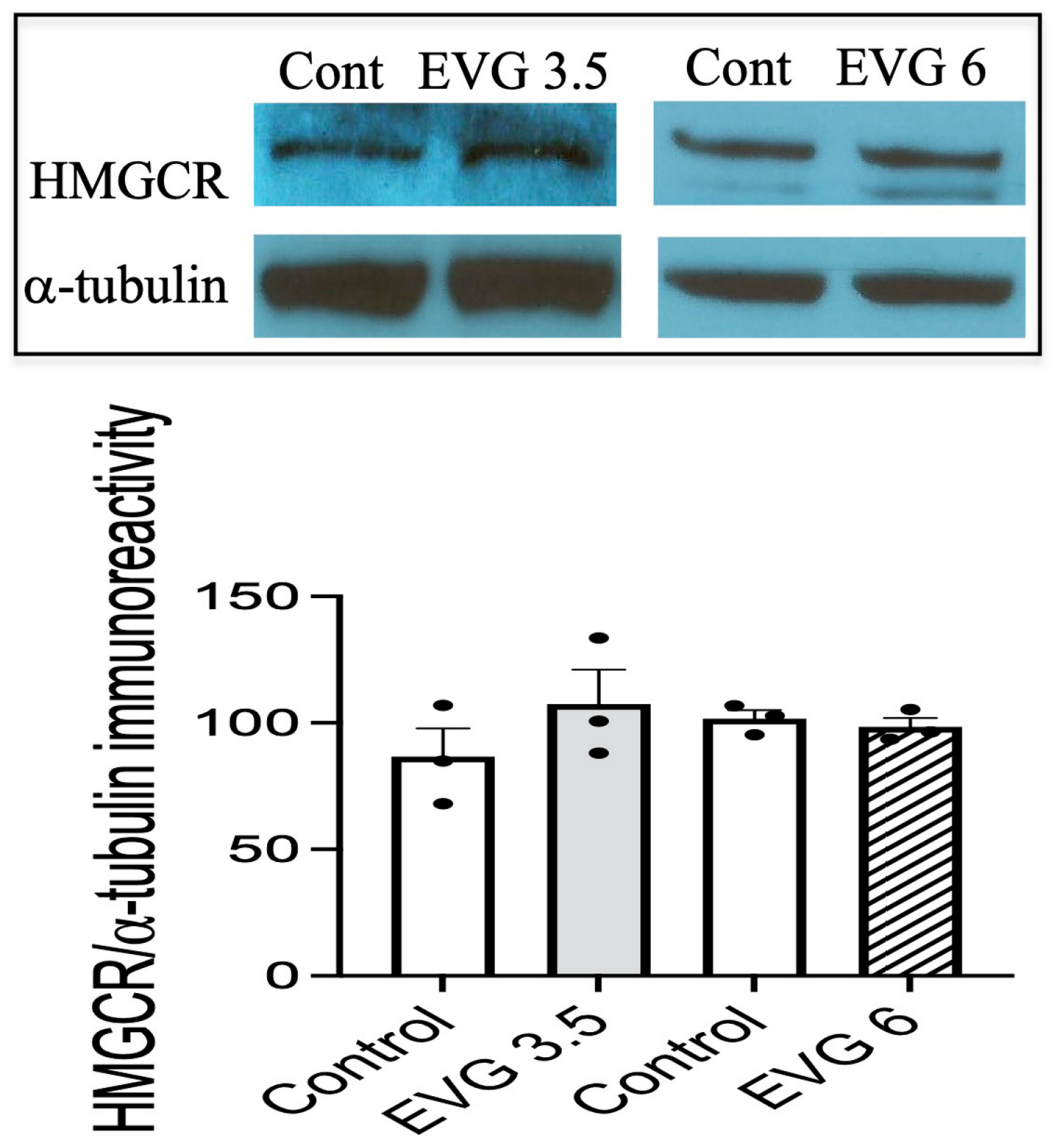
Protein expression of hydroxy-methyl-glutaryl CoA reductase (HMGCR) is not affected by EVG exposure to oligodendrocyte cultures. Oligodendrocyte cultures grown for 3 days in a differentiation medium with EVG (3.5 or 6 μM) or without (Control) were subjected to Western blot analysis and quantification of band intensities of HMGCR protein immunoreactivities normalized to α-tubulin as loading control. Graph data are expressed as mean ± SEM from three independent cell culture preparations.

The myelin membrane contains a high level of cholesterol, and its availability is a rate-limiting factor. Therefore, we measured *de novo* synthesis of cholesterol following EVG exposure (3.5 or 6 μM) of oligodendrocyte cultures differentiated for 3 days. Cholesterol synthesis was not affected by either doses of EVG (controls: 30.7 μg/mg protein ±4.41 versus 3.5 μM EVG-treated cells: 34.5 μg/mg protein ±5 versus 6 μM EVG-treated cells: 26.7 μg/mg protein ±1.73; *n* = 3). In addition, protein levels of hydroxy-methyl-glutaryl CoA reductase (HMGCR), the rate-limiting enzyme for cholesterol synthesis, did not differ between controls and EVG-treated cells after 3 days of differentiation (Figure 6).

### 3.7 SREBP-2 protein and mRNA expressions are altered in the presence of EVG

**Figure 7:**
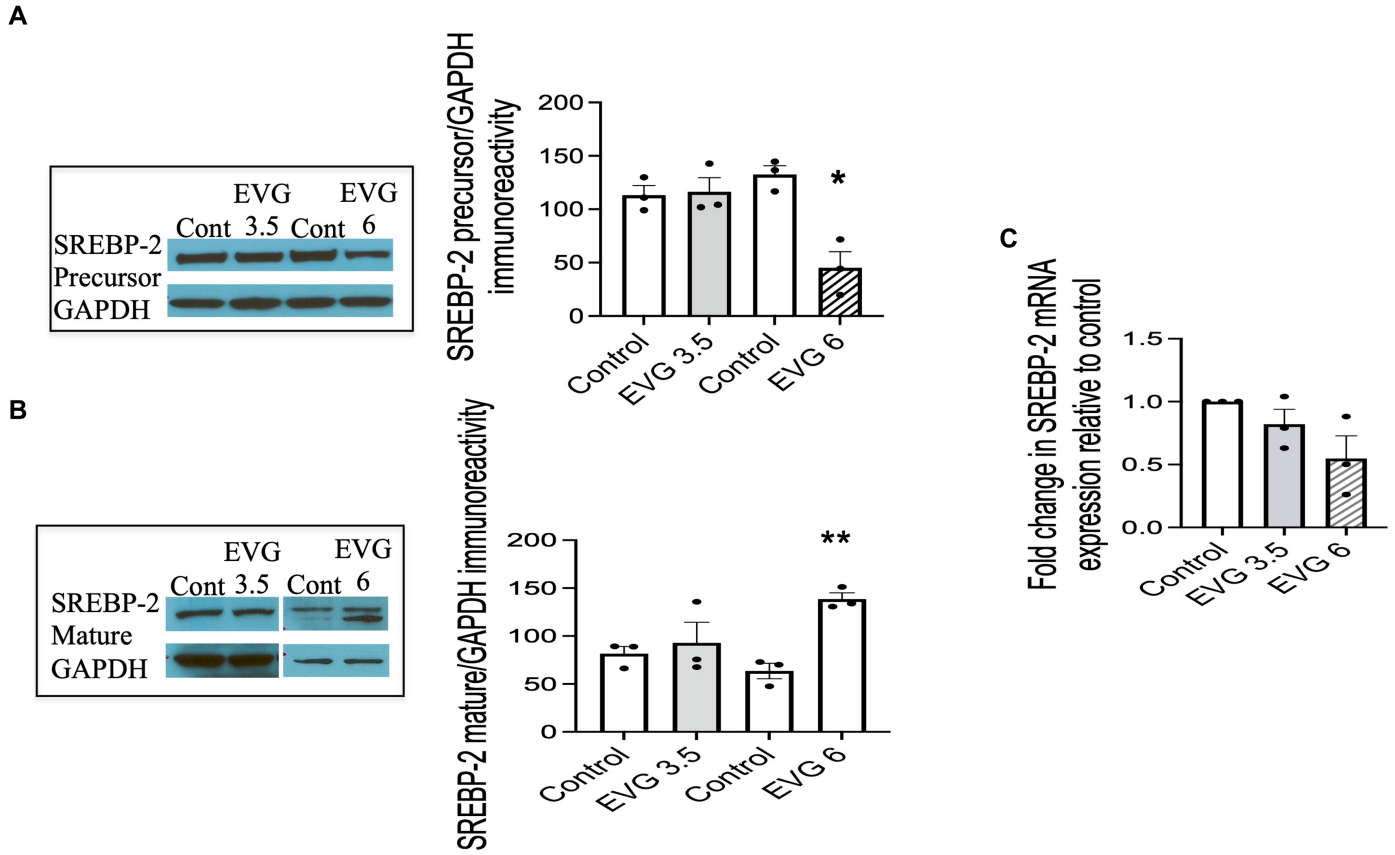
EVG alters the protein expression of sterol regulatory element-binding protein 2 (SREBP-2) in oligodendrocyte cultures. Oligodendrocyte cultures were grown in a differentiation medium with EVG (3.5 or 6 μM) or without (Control). After 3 days, cells were harvested and prepared for immunoblotting or qRT-PCR. **(A,B)** Western blots and quantification of band intensities of SREBP-2 precursor **(A)** and mature form **(B)** normalized to GAPDH as loading control. Graph data are expressed as mean ± SEM from three independent cell culture preparations. *p < 0.05 versus control; **p < 0.01 versus control. In **(C)**, the graph shows mRNA expression for SREBP-2 relative to the control. Graph data are expressed as mean ± SEM from three independent cell culture preparations.

To determine whether EVG exposure also affected the processing of SREBP-2, oligodendrocyte cultures were differentiated for 3 days with or without EVG (3.5 or 6 μM), before cell extracts were collected and processed for Western blotting. We found that the protein level of the SREBP-2 precursor form was markedly decreased at 6 μM compared with controls (Figure 7A). The mature form was also altered by 6 μM EVG, resulting in a stronger lower band of the doublet compared with controls (Figure 7B). qRT-PCR analysis performed on differentiating oligodendrocyte cultures for 3 days showed SREBP-2 mRNA levels trended lower with increasing EVG concentrations, but this did not reach statistical significance (Figure 7C). Although the presence of EVG also disrupts the expression of SREBP-2, it does not seem to be affecting the cholesterol pathway.

### 3.8 The integrase strand transfer inhibitor (INSTI) raltegravir (RAL) does not alter SREBP expression

**Figure 8:**
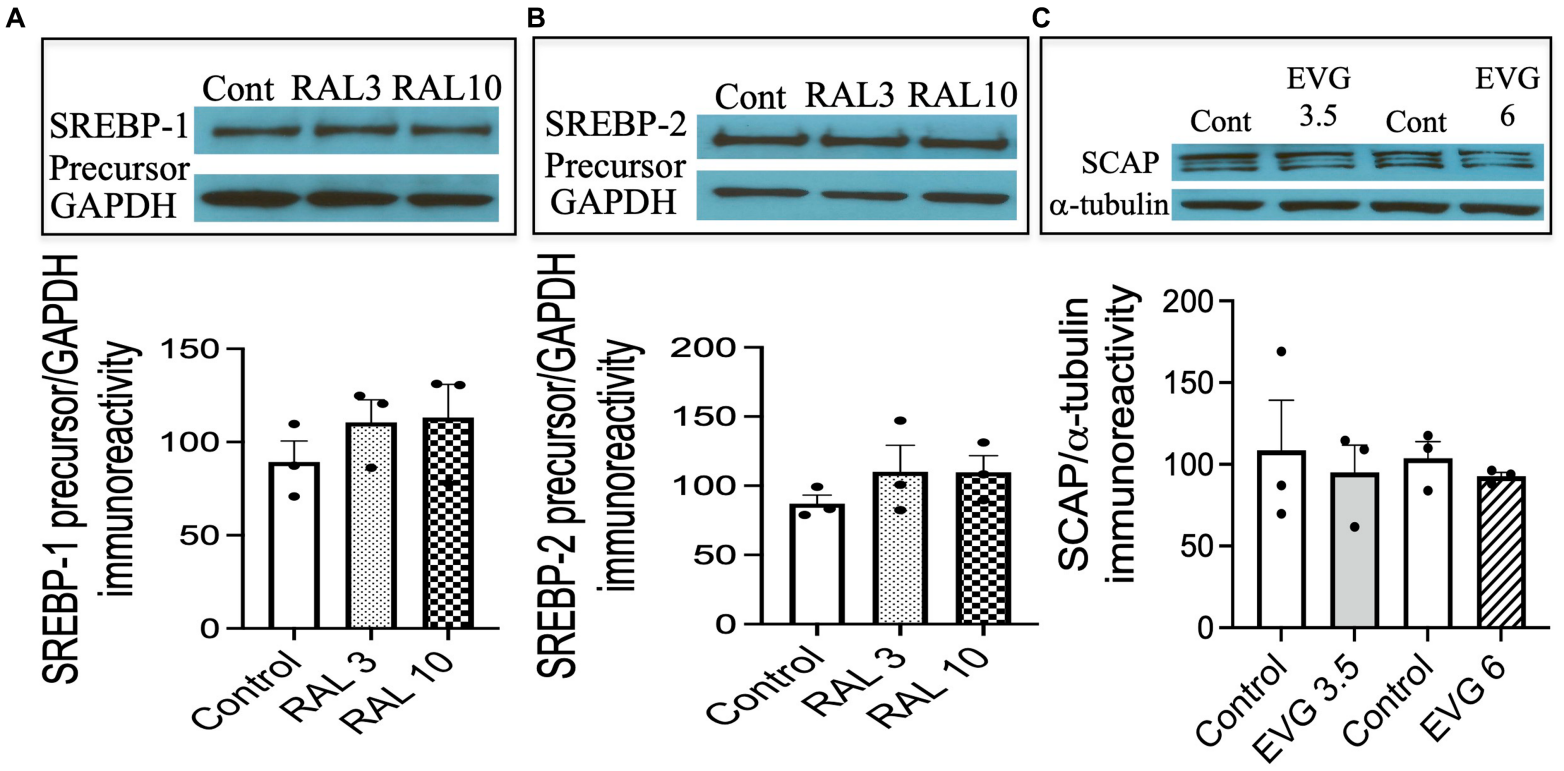
Integrase strand transfer inhibitor (INSTI) raltegravir (RAL) does not affect the expression of SREBPs in oligodendrocyte cultures, while the protein expression of SREBP cleavage activating protein (SCAP) is not affected by EVG exposure to oligodendrocyte cultures. **(A,B)** Show Western blots and quantification of band intensities of RAL-exposed oligodendrocyte cultures grown in a differentiation medium for 3 days with RAL (3 or 10 μM) or without (Control) and probed with anti-SREBP-1 **(A)** and anti-SREBP-2 **(B)**. GAPDH was used as a loading control. Graph data are expressed as mean ± SEM from two to three independent cell culture preparations. **(C)** Oligodendrocyte cultures grown for 3 days in a differentiation medium with EVG (3.5 or 6 μM) or without (Control) were subjected to Western blot analysis and quantification of band intensities of SCAP protein immunoreactivities normalized to α-tubulin as loading control. Graph data are expressed as mean ± SEM from three independent cell culture preparations.

To assess whether the effect of an INSTI compound that did not inhibit oligodendrocyte differentiation altered SREBP-1 and SREBP-2 expression as well, we tested the effect of RAL, another INSTI recommended as part of initial regimens for persons with HIV. We had previously shown RAL to have no effect on oligodendrocyte maturation or myelin protein expression (Roth et al., 2020). Cells were switched to a differentiation medium and incubated with or without RAL (3 or 10 μM) for 3 days, before cell extracts were processed for Western blot analysis. As shown in Figure 8, RAL exposure did not alter SREBP-1 or SREBP-2 precursor levels, compared to controls (Figures 8A,B). These data indicate that INSTIs may have differential effects on oligodendrocyte maturation.

### 3.9 The expression of SREBP-cleavage activating (SCAP) protein is not altered by EVG exposure

Next, we asked whether SCAP, an SREBP activator essential for myelin lipid synthesis, could be affected by EVG exposure. OPC cultures were differentiated for 3 days in the presence of EVG (3.5 or 6 μM) before cell extracts were processed for Western blot analysis. SCAP expression was not affected following EVG treatment of oligodendrocyte cultures (Figure 8C).

### 3.10 The integrated stress response (ISR) is minimally implicated in EVG-mediated alteration in SREBP expression

**Figure 9:**
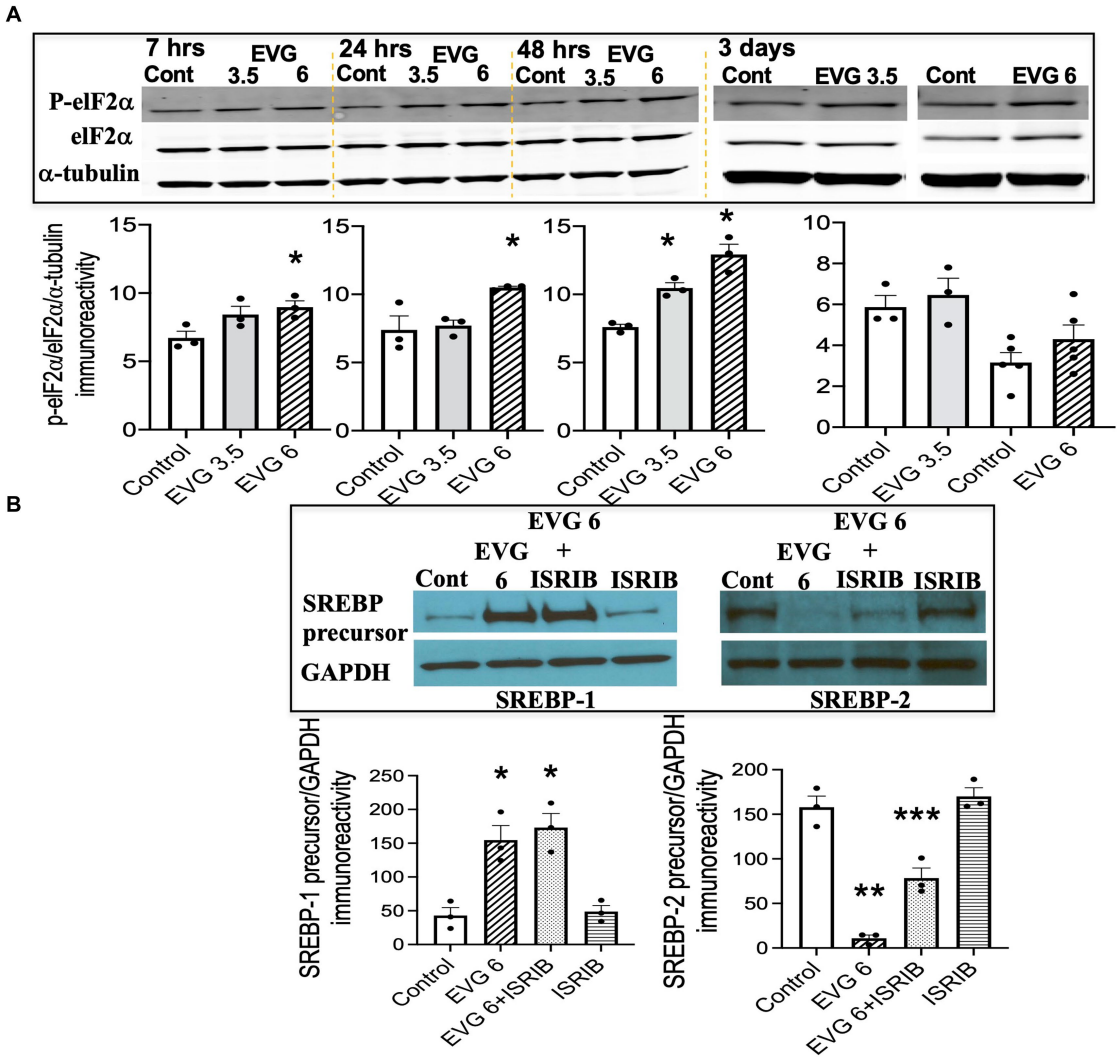
EVG triggers the integrated stress response (ISR) in oligodendrocyte cultures, but the integrated stress response inhibitor (ISRIB) minimally prevents EVG-induced SREBP alterations. Oligodendrocyte cultures were grown in a differentiation medium with EVG (3.5 or 6 μM) or without (Control), plus or minus the ISR-inhibitor ISRIB (5 μM). In **(A)**, cells were harvested at 7, 24, and 48 h or 3 days and prepared for immunoblotting, and band intensities were quantified for phosphorylated elF2α (P-elF2α), elF2α, and α-tubulin immunoreactivities. P-elF2α bands were normalized to elF2α and then to α-tubulin as loading control. Graph data are expressed as mean ± SEM from three independent cell culture preparations. *p < 0.05 versus control. In **(B)**, cells were grown in differentiation medium for 3 days with EVG (6 μM) or without (Control), plus or minus the ISR inhibitor ISRIB (5 μM). Western blots and quantification of band intensities were performed for lysates probed with anti-SREBP-1 or anti-SREBP-2. GAPDH was used as a loading control. Graph data are expressed as mean ± SEM from three to five independent cell culture preparations. *p < 0.05 versus control; **p < 0.01 versus control; ***p < 0.05 versus EVG 6 μM.

The findings that exposure to oligodendrocyte cultures to EVG led to decreased protein expressions of myelin proteins as well as key enzymes in the fatty acid pathway may result from attenuation of protein translation triggered by activation of the ISR. In addition, we previously reported evidence that EVG treatment of oligodendrocyte or neuroglial cultures activated the ISR (Stern et al., 2018; Roth et al., 2020), and Harding et al. (2005) demonstrated that stimulation of the ISR decreased SREPB activation. The ISR is a cytoprotective mechanism that is triggered under various stress conditions to maintain cellular proteostasis (Pakos-Zebrucha et al., 2016). The focal point of ISR induction is the phosphorylation of eukaryotic translation initiation factor 2 alpha (elF2α), which leads to a reduction in global protein synthesis while selectively promoting the translation of protective proteins and transcription factors. Therefore, to determine whether EVG-induced alteration in SREBP expression involved the ISR, we assessed the phosphorylation level of elF2α following EVG treatment of oligodendrocyte cultures. Cells were allowed to differentiate for 7 h to 3 days with or without EVG (3.5 or 6 μM), before cells were harvested for Western blot analysis. Figure 9A shows the time course of elF2α phosphorylation: At 7 h post-initiation of differentiation, phosphorylated elF2α was significantly increased by the addition of 6 μM EVG while it trended higher at 3.5 μM, compared with controls. While the phosphorylation levels continued to increase between 24 h and 48 h in 6 μM EVG-treated cells, it was only increased at 48 h in cells exposed to 3.5 μM EVG, compared with controls. After 3 days of differentiation, elF2α phosphorylation in cells exposed to 6 μM EVG was not significantly higher than in controls. We next asked whether the ISR inhibitor ISRIB (Sidrauski et al., 2013) would prevent the effect of EVG on SREBP expression in oligodendrocyte cultures. OPCs were pre-treated with ISRIB (5 μM) for 2 h before cells were switched to ISRIB-containing differentiation medium and allowed to differentiate for 3 days with or without 6 μM EVG, at which time cell extracts were collected for Western blotting. ISRIB did not block the EVG-mediated increase in SREBP-1 precursor protein level, although it attenuated the drastic decrease in SREBP-2 precursor protein expression that was observed after EVG exposure alone (Figure 9B). Higher ISRIB concentrations (up to 20 μM) did not improve its effect further.

### 3.11 Addition of serum albumin, but not fatty acids, to oligodendrocyte cultures prevents the EVG-mediated alteration in oligodendrocyte differentiation

**Figure 10:**
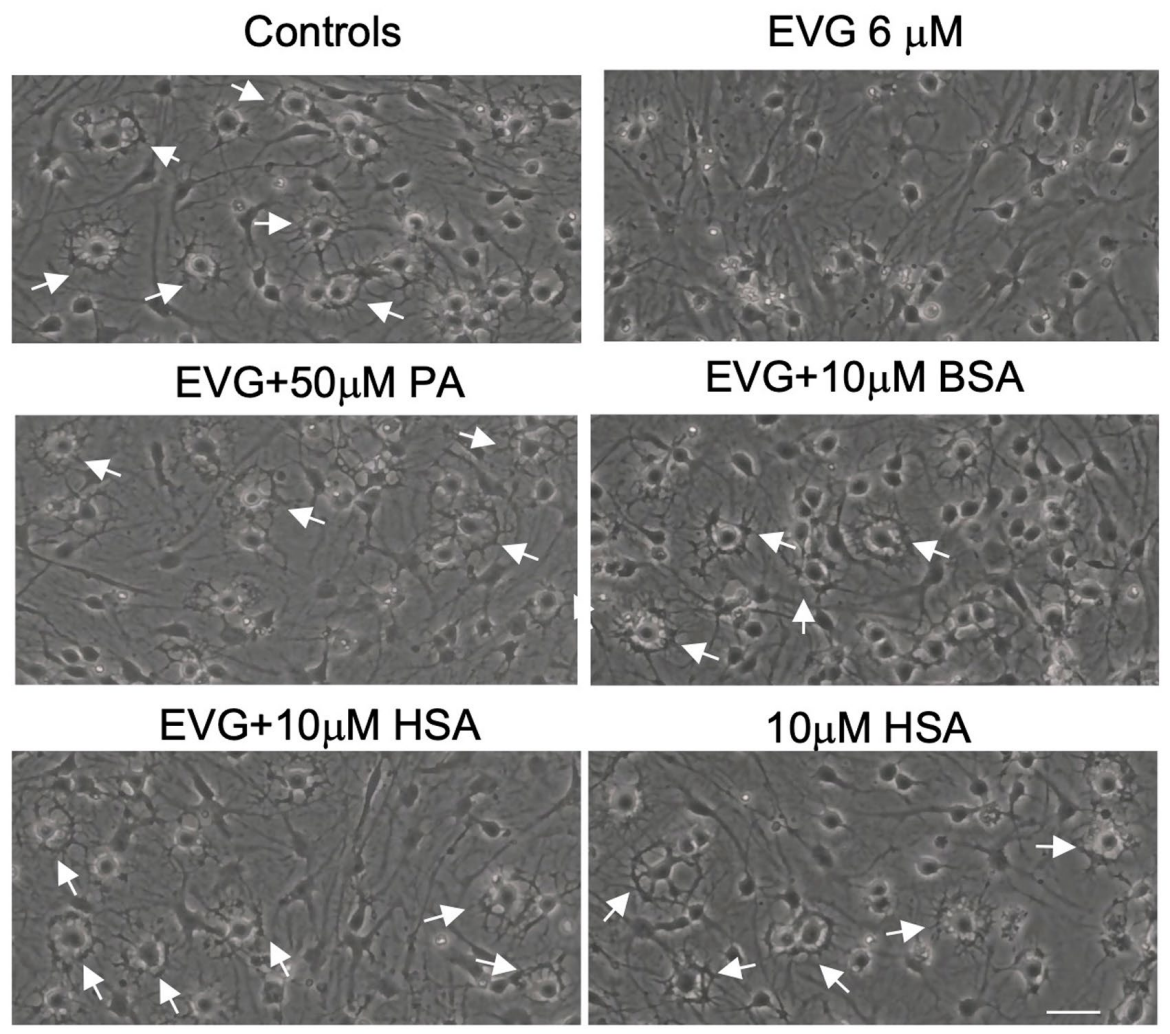
Serum albumin protects against the EVG-induced inhibition of oligodendrocyte differentiation in oligodendrocyte cultures. Photomicrographs of sister cultures after 3 days of oligodendrocyte differentiation taken under phase-contrast microscopy. Control cultures display numerous “halo” cells representative of differentiating oligodendrocytes with processes wrapped around their cell body (arrows). Cultures exposed to EVG (6 μM) for 3 days do not show “halo” cells, and process growth is reduced. Cultures co-treated with bovine (BSA) or human (HSA) serum albumins exhibit many “halo” cells as in controls (arrows) as do cultures in which BSA-containing palmitate (PA) has been added to the culture medium. Scale bar: 35 μm.

**Figure 11:**
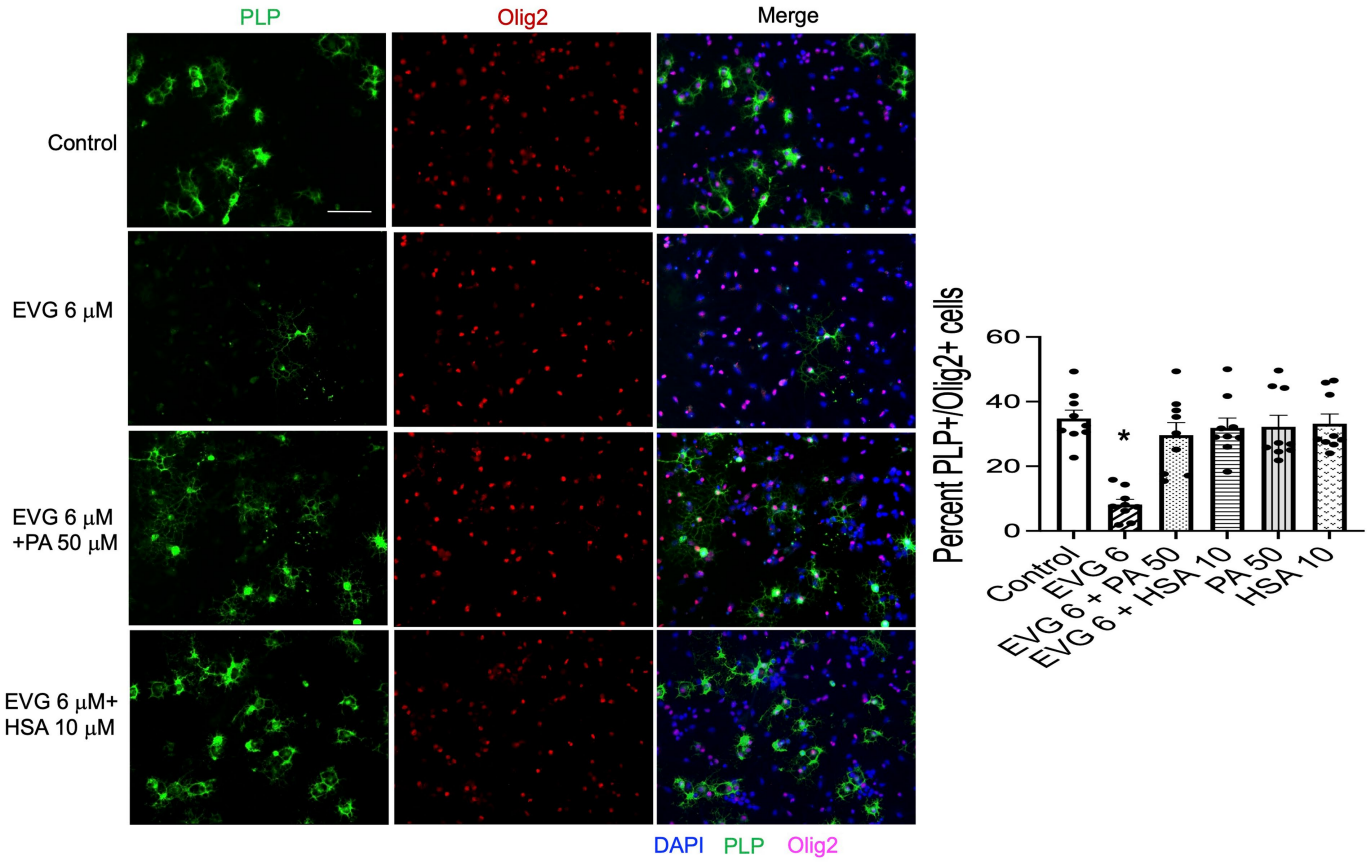
Serum albumin protects against the EVG-induced inhibition of oligodendrocyte differentiation in oligodendrocyte cultures. Representative images of PLP immunostaining in oligodendrocyte cultures grown for 3 days in a differentiation medium with EVG (6 μM), with EVG and PA/BSA (50 μM), with EVG and HSA (10 μM), or without (Control). Cells were triple-labeled for DAPI to stain nuclei (blue), PLP to label differentiating oligodendrocytes (green), and Olig2 to label oligodendrocyte lineage cells (pink). Scale bar: 50 μm. The graph represents the number of PLP-positive cells normalized to the number of Olig2-expressing cells. *p < 0.0005 versus control, EVG 6 + PA50 and EVG 6 + HSA10 (ANOVA).

The significant reduction in palmitate (PA) synthesis we observed following EVG exposure prompted us to test whether adding PA to the culture medium could counteract the effect of EVG. OPC cultures were switched to a differentiation medium containing PA conjugated to BSA (PA/BSA) at various concentrations (20–100 μM) in the presence of EVG 6 μM for 3 days, including a condition with PBS/BSA added as a control. Co-treatment of cultures with both PA/BSA and PBS/BSA improved oligodendrocyte differentiation as observed by examining cell morphology under phase-contrast microscopy; in particular, many cells formed processes wrapped around their cell body, a “halo” typical of many differentiating oligodendrocytes, similar to control cultures (Figure 10). These “haloed” cells were consistently absent from cultures treated with EVG alone. To determine whether fatty acids themselves could mitigate the effect of EVG on oligodendrocyte differentiation, we treated oligodendrocyte cultures with a mixture of fatty acids in a BSA-free medium and examined the impact on cell morphology. Cells treated with various concentrations of fatty acids together with EVG were not morphologically different from those exposed to EVG alone, after 3 days of differentiation (data not shown). To ascertain serum albumin’s protective role and establish its specificity, we incubated the cells with 10 μM human recombinant serum albumin (HSA) in the presence of 6 μM EVG and differentiated them for 3 days. As we previously observed with BSA alone, in HSA-treated cultures many oligodendrocytes exhibited the typical differentiating “halo” morphology under phase-contrast microscopy, regardless of the presence or absence of EVG (Figure 10). To confirm that HSA treatment improved oligodendrocyte maturation in the presence of EVG, cells were differentiated for 3 days and then immunostained for proteolipid protein (PLP) and Olig2. As shown previously, 6 μM EVG greatly decreased the number of PLP-expressing oligodendrocytes compared with controls. However, the decrease in PLP expression was prevented by co-incubating with either 50 μM PA/BSA or 10 μM HSA, indicating that HSA alone was sufficient to protect against the effect of EVG on PLP expression (Figure 11).

### 3.12 Addition of serum albumin prevents the EVG-mediated increase in SREBP-1 expression in differentiating oligodendrocyte cultures

**Figure 12:**
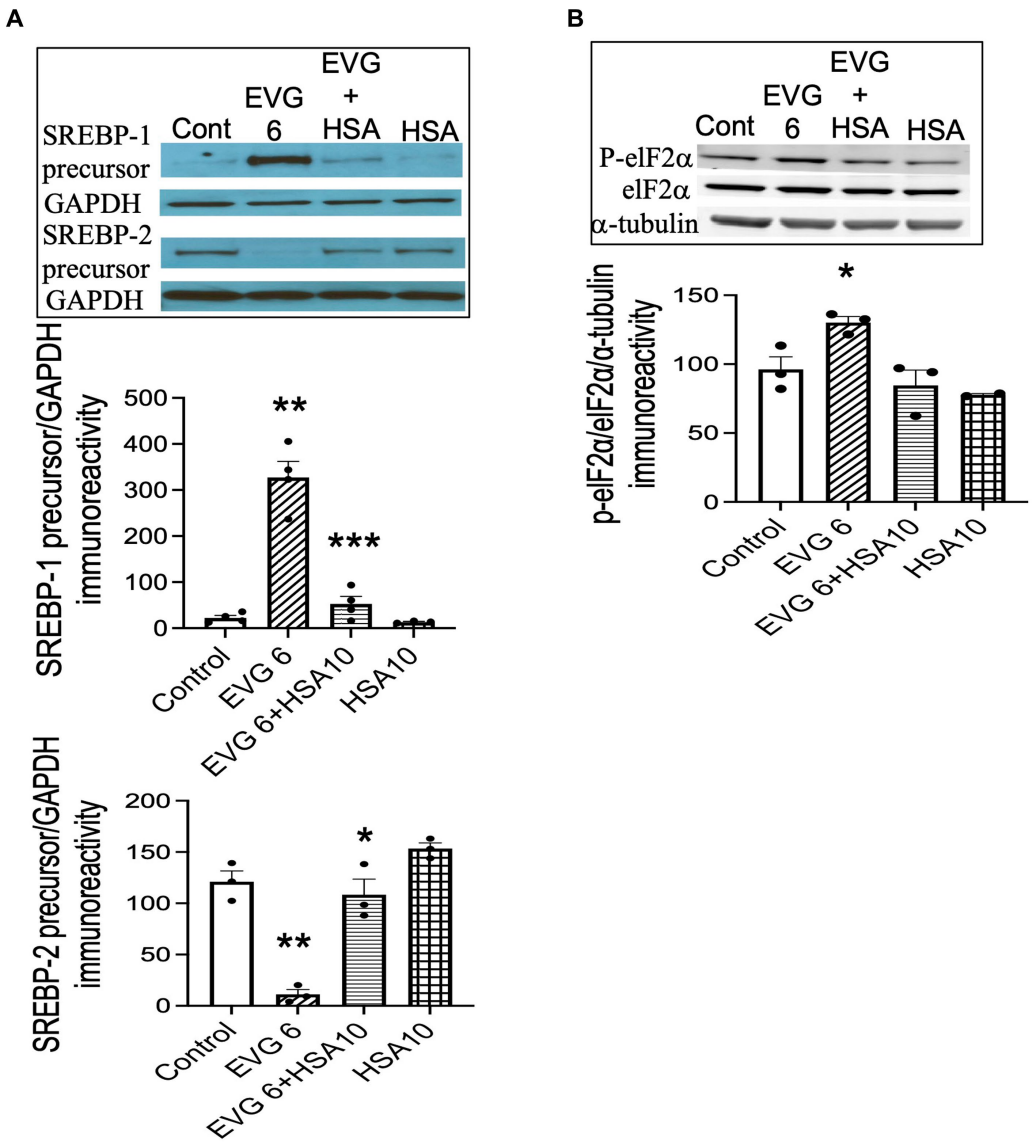
Serum albumin protects against the EVG-induced alteration in SREBP expression and prevents the increase in elF2α phosphorylation in oligodendrocyte cultures. **(A)** Oligodendrocyte cultures were grown in a differentiation medium with EVG (6 μM), EVG + 10 μM HSA, 10 μM HSA alone, or without (Control). After 3 days, cells were harvested and prepared for immunoblotting. Western blots and quantification of band intensities for SREBP-1 and SREBP-2 normalized to GAPDH as loading control. SREBP-1: **p < 0.005 versus control; ***p < 0.005 versus EVG 6 μM. SREBP-2: *p < 0.05 versus EVG 6 μM; **p < 0.005 versus control. **(B)** Western blot and quantification of band intensities for phosphorylated elF2α (P-elF2α), elF2α, and α-tubulin immunoreactivities. P-elF2α bands were normalized to elF2α and then to α-tubulin as loading control. Graph data are expressed as mean ± SEM from three independent cell culture preparations. *p < 0.05 versus control and EVG 6 μM + HSA 10 μM.

**Figure 13:**
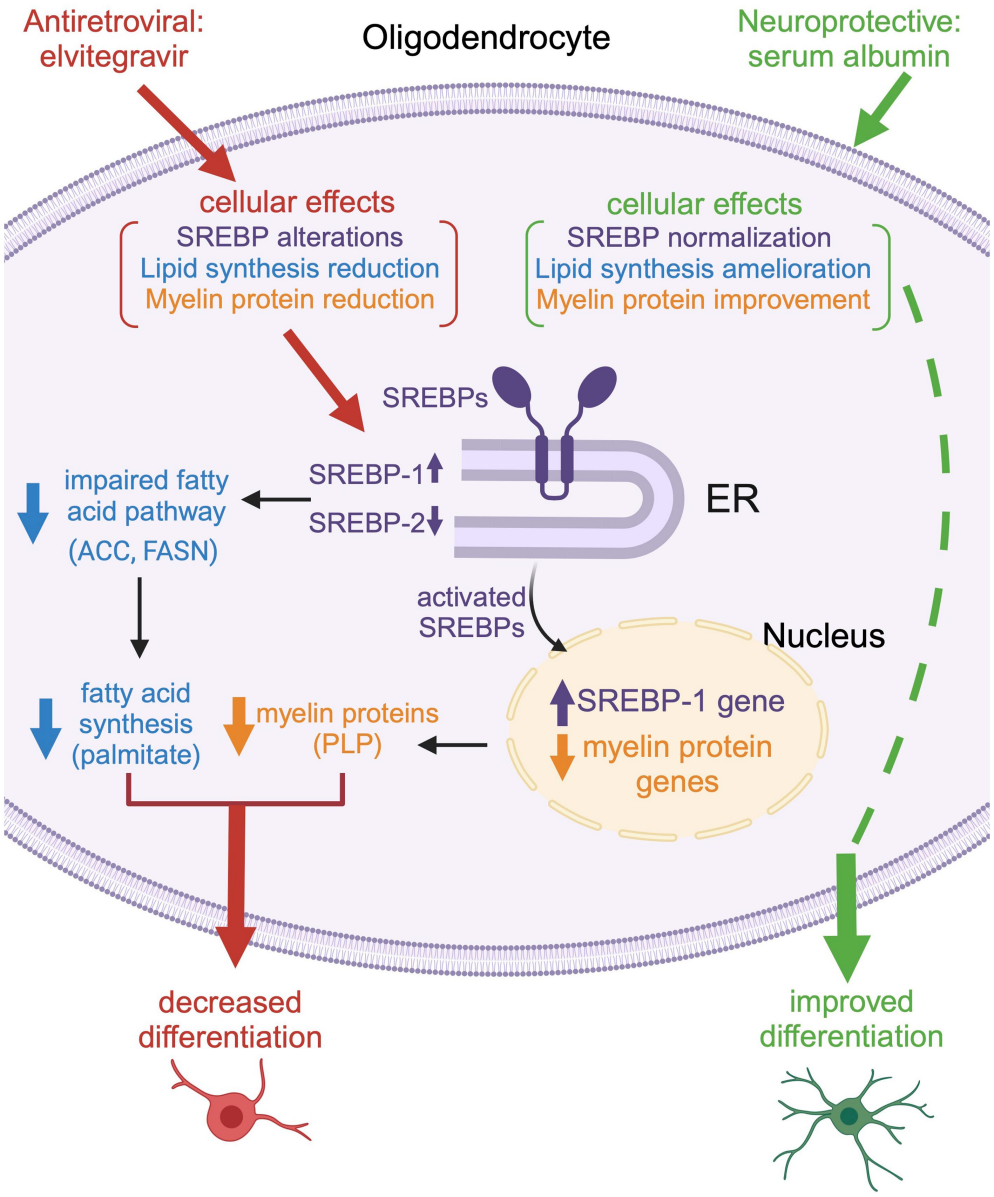
Schematic representation of the effect of EVG on oligodendrocyte maturation. Exposure to EVG alters SREBP processing (SREBP-1 is increased, while SREBP-2 is decreased), and the expression of myelin protein genes is decreased, which results in reduced myelin protein expression [such as proteolipid protein (PLP)]. In addition, the protein expression of lipid enzymes in the fatty acid pathway [acetyl-CoA carboxylase (ACC) and fatty acid synthase (FASN)] is diminished. This leads to a reduction in fatty acid synthesis (palmitate) and impaired differentiation of oligodendrocytes. Co-treatment with serum albumin mitigates the effect of EVG by ameliorating SREBP processing and restoring myelin protein expression, leading to improved oligodendrocyte maturation. However, fatty acid synthesis is not completely reestablished, suggesting that other cellular functions may remain dysregulated. Reproduced with permission from C. Long. Created with BioRender.com.

Our data showed that both BSA and HSA protected against the EVG-induced alteration in oligodendrocyte maturation. To determine whether this effect also applied to the dramatic changes we observed in SREBP expression, EVG-exposed OPC cultures were differentiated for 3 days with or without 10 μM HSA, before cell extracts were analyzed by Western blotting. Figure 12A shows that the EVG-mediated increase in SREBP-1 precursor expression was almost completely abolished by HSA co-incubation. In addition, the EVG-induced dramatic reduction in SREBP-2 precursor expression was greatly attenuated by HSA co-incubation. The EVG-induced increase in ISR activity prompted us to determine whether co-incubation with HSA would prevent the phosphorylation of elF2α. As shown in Figure 12B, cells treated with 6 μM EVG + 10 μM HSA for 48 h (corresponding to peak elF2α phosphorylation, see Figure 9A) did not show an increase in phosphorylated elF2α compared with EVG-exposed cells, suggesting that the ISR was not activated in the presence of HSA. Finally, since HSA co-treatment improved oligodendrocyte maturation, we assessed whether the EVG-induced decrease in PA synthesis could also be reversed. Quantification of *de novo* PA synthesis revealed that EVG-treated cultures exposed to HSA synthesized 40% more PA than cultures incubated with EVG alone. Indeed, PA synthesis was reduced by 60% in EVG-treated cells compared with controls, while a 36% reduction was observed in EVG + HSA-treated cells (*n* = 3). Although HSA treatment substantially ameliorated the changes in myelin protein expression and greatly reduced the EVG-induced alteration in SREBP processing, PA synthesis remained affected, indicating that additional mechanisms regulating lipid production remain dysregulated despite the impact of HSA (Figure 13).

## 4 Discussion

Persistent white matter damage is often observed in persons with HIV on antiretroviral therapy. Although HIV infection itself can cause white matter injury, antiretroviral toxicity may contribute to or prolong myelin damage by altering oligodendrocyte maturation and myelin formation and/or maintenance (Jensen et al., 2015; Roth et al., 2020; Festa et al., 2021, 2023). This, in turn, may help explain at least in part, the high prevalence of HAND among persons with HIV receiving antiretroviral therapy because myelin integrity is essential for normal brain function. Our present data further establish such a possibility. We show that the integrase strand transfer inhibitor EVG prevented oligodendrocyte differentiation by reducing the synthesis of myelin proteins while disrupting SREBP processing, which was accompanied by a dysregulation of the transcription of SREBPs and their protein expression as well as that of target genes of the lipid pathway. These biochemical alterations resulted in dramatically reduced fatty acid synthesis in EVG-exposed cells. Perturbations in lipid metabolism during myelination may have deleterious repercussions for persons with HIV; therefore, improving our understanding of the effects of antiretrovirals in the context of white matter damage may help devise therapeutic strategies that will significantly reduce their impact on brain function.

We show that EVG exerted its effect at every stage of oligodendrocyte differentiation, as evidenced by decreased expressions of early, intermediate, and late stage maturation markers. In addition, mRNA expression of the mature marker proteolipid protein (PLP) was also decreased, suggesting that EVG interfered with the maturation process at the transcriptional level. Alteration of oligodendrocyte differentiation has been reported for several antiretroviral drugs in the protease inhibitor class (Jensen et al., 2015; Festa et al., 2021) and the integrase strand transfer inhibitor (INSTI) class (Roth et al., 2020; Festa et al., 2023). We previously demonstrated that the INSTI raltegravir (RAL) did not inhibit oligodendrocyte differentiation (Roth et al., 2020), and here, we show that the expression of SREBP-1 and SREBP-2 in oligodendrocytes was not affected by RAL treatment. Taken together, these data indicate that there is a within-class differential effect among antiretrovirals on oligodendrocyte differentiation, which should be considered when devising regimens for persons with HIV.

The effect of EVG on SREBP protein levels revealed an opposite pattern of alterations: SREBP-1 precursor protein expression was increased while that of SREBP-2 precursor was decreased, compared with controls. Differential effects between SREBP-1 and SREBP-2 mRNA and/or protein levels have been shown *in vivo* in hamsters fed a high-fat diet (Singh et al., 2016). In this study, SREBP-2 mRNA expression was reduced whereas that of SREBP-1 was elevated by the hyperlipidemic diet. In addition, the level of SREBP-2 precursor protein was also decreased. While presently the level of SREBP-2 mRNA trended lower with increasing doses of EVG compared with controls, it is unlikely that it accounted for the dramatic decrease in SREBP-2 precursor protein abundance observed after EVG exposure. It is possible that during EVG exposure, the turnover rate of the precursor is increased or that enhanced degradation of the protein is taking place. SREBPs are regulated by multiple cellular mechanisms, some of them affecting their proteolytic processing, which may be altered by EVG and which could impair expression levels of the protein (DeBose-Boyd and Ye, 2018). While 6 μM EVG led to the appearance of a stronger lower SREBP-2 mature band compared with controls, the significance of this observation is unclear. It is possible that the lower fragment resulted from proteolytic cleavage of the mature form or that degradation of the mature form may be impaired by EVG exposure, leading to its accumulation. Nevertheless, a drastic reduction in SREBP-2 precursor protein expression that did not correlate with a change in mature form was also observed following site-1 protease inhibition in oligodendrocyte cultures (Monnerie et al., 2017).

The higher SREBP-1 precursor level observed following EVG treatment may reflect the large increase in SREBP-1 mRNA level. Interestingly, elevated SREBP-1 mRNA expression has been reported in HIV-1 transgenic rats after they were given combined antiretroviral therapy (cART) orally (ElZohary et al., 2019). It is not clear at present why fatty acid synthesis is reduced when SREBP-1 levels are increased. Lipid enzymes may be regulated independently, at the translational and/or post-translational levels, in addition to altered expression and enzymatic activity. For example, decreased fatty acid synthase (FASN) expression accompanied by reduced palmitate synthesis that occurred in the absence of SREBP-1 alteration was reported in oligodendrocytes by Lebrun-Julien et al. (2014). Another study in which treatment of cancer cells with the HIV protease inhibitor nelfinavir showed increased SREBP-1 levels correlated with decreased expression of FASN (Guan et al., 2012). These examples illustrate the complexity of SREBP regulation that involves multiple cellular processes, some of which are likely to be altered by EVG.

The EVG-induced alteration in SREBP expression paralleled a decrease in acetyl-CoA carboxylase (ACC) and FASN protein levels and most likely caused the perturbation in lipid metabolism we observed. This effect was not preceded by a change in transcription in either ACC or FASN genes; however, a non-linear correlation between gene expression level and abundance of the protein is not unusual (Liu et al., 2016). In contrast, hydroxy-methyl-glutaryl CoA reductase (HMGCR) protein level was not reduced, and cholesterol synthesis was not affected by EVG treatment. These findings suggest that EVG impacts fatty acid metabolism independent of changes in cholesterol synthesis.

SREBPs are major regulators of cellular lipid metabolism, and myelination requires oligodendrocytes to elaborate tremendous amounts of lipids; any dysregulation in lipid synthesis may impair oligodendrocyte maturation by affecting the transcription, expression, transport, and/or localization of specific myelin proteins such as myelin basic protein (MBP) and proteolipid protein (PLP) (Simons et al., 2000; Maier et al., 2009; Saher et al., 2009). Furthermore, fatty acids are the structural backbone of most lipids incorporated into myelin. As such, loss of SREBP-mediated lipid synthesis has been correlated with hypomyelination in mice, highlighting the importance of SREBPs in myelin formation (Verheijen et al., 2009; Camargo et al., 2017).

We found that EVG exposure increased the phosphorylation of elF2α, indicating that the integrated stress response (ISR) was activated. This is consistent with previous reports showing that EVG treatment elicited a higher level of phosphorylated elF2α in both oligodendrocyte and neuroglial cultures (Stern et al., 2018; Roth et al., 2020). The potent ISR inhibitor ISRIB, which prevents the effects of elF2α phosphorylation (Sidrauski et al., 2013), only moderately counteracted the effect of EVG on the expression of SREBP-2 (Figure 8B), suggesting that other cellular pathway(s) may be involved. Stern et al. (2018) also reported a mild attenuation of ISRIB pretreatment on EVG-induced toxicity in primary rat cortical neuroglial cultures, an indication that the ISR is not the only pathway disrupted by EVG.

We showed that human serum albumin (HSA) protected oligodendrocyte cultures from the deleterious effect of EVG on OPC differentiation and SREBP processing. It also prevented the phosphorylation of elF2α, indicating that the ISR was not activated in the presence of HSA. Albumin’s neuroprotective action has been reported *in vitro* in preventing neurotoxicity in hippocampal slice cultures (Dani et al., 2019) and DNA damage (Baltanas et al., 2009), zinc toxicity (Lin et al., 2005), oxidative stress (Gum et al., 2004), and intracellular calcium overload (Gallego-Sandin et al., 2005) in cultured neurons. Its beneficial effect has also been documented *in vivo* in rat (Park et al., 2017; Yildirim et al., 2018) and in humans (Palesch et al., 2006; Suarez et al., 2012). Interestingly, in a transgenic mouse model of Alzheimer’s disease (Ezra et al., 2016), HSA-treated mice showed increased myelin integrity and myelin basic protein (MBP) protein level. It remains unclear whether the effect of HSA in our system is direct or secondary. Although a receptor for albumin has been found in astrocytes, it is not known whether oligodendrocytes also express albumin receptors. However, specific uptake mechanisms may exist through which albumin could exert its metabolic effects, as reported for neurons, astrocytes, and microglia (Ivens et al., 2007; Van Vliet et al., 2007; Braganza et al., 2012). Alternatively, while EVG interaction with serum albumin present in the culture medium may differ from that exists in human plasma, albumin may bind EVG in our culture conditions because EVG has a high affinity for plasma proteins, which could mitigate the effect of EVG on oligodendrocyte differentiation.

Several studies have established a link between SREBP gene expression changes and myelin alterations: There is genetic evidence supporting the involvement of the SREBP genes in schizophrenia, a psychiatric disorder with well-described white matter abnormalities (Steen et al., 2017). In bipolar disorder, SREBP-2 gene polymorphism has been linked to white matter microstructure differences, a major characteristic of the disease involving all the main white matter tracts (Poletti et al., 2016). Furthermore, a study analyzing gene expression in white matter from persons with HIV on combined antiretroviral therapy (cART) reported not only a decline in both SREBP-1 and SREBP-2 gene expression but also in that of oligodendrocyte myelin genes, when compared with HIV-negative controls (Solomon et al., 2019). HIV infection has been associated with lower SREBP-2 gene expression in monocytes derived from cART-naive persons with HIV than in HIV-negative controls, while the cART-treated/HIV-positive group displayed an even stronger decrease (Feeney et al., 2013). The present study shows that exposure to oligodendrocyte cultures to EVG altered SREBP expression, leading to decreased fatty acid synthesis. Since most myelin lipids are structurally fatty acid-based, reduced incorporation of fatty acids into the myelin membrane may have serious consequences by changing its physical characteristics, altering its fluidity, and affecting membrane protein functions. Such disruption together with diminished myelin protein synthesis may affect the formation and maintenance of myelin and strongly suggests the possibility that white matter damage in persons with HIV may be exacerbated by cART.

## Data availability statement

The raw data supporting the conclusions of this article will be made available by the authors, without undue reservation.

## Ethics statement

The animal study was approved by the Children’s Hospital of Philadelphia Institutional Animal Care and Use Committee (IACUC). The study was conducted in accordance with the local legislation and institutional requirements.

## Author contributions

HM: Writing – original draft, Writing – review & editing. MR: Writing – review & editing. LR: Writing – review & editing. CL: Writing – review & editing. JM: Writing – review & editing. KJ-S: Writing – review & editing. JG: Writing – review & editing.
